# Effectiveness of Emotional Memory Reactivation vs Control Memory Reactivation Before Electroconvulsive Therapy in Adult Patients With Depressive Disorder: A Randomized Clinical Trial

**DOI:** 10.1001/jamanetworkopen.2020.12389

**Published:** 2020-08-04

**Authors:** Dominique S. Scheepens, Jeroen A. van Waarde, Freek ten Doesschate, Mirjam Westra, Marijn C. W. Kroes, Aart H. Schene, Claudi L. H. Bockting, Robert A. Schoevers, Damiaan A. J. P. Denys, Henricus G. Ruhé, Guido A. van Wingen

**Affiliations:** 1 Department of Psychiatry, Amsterdam Neuroscience, Amsterdam UMC, University of Amsterdam, Amsterdam, the Netherlands; 2 Department of Psychiatry, Rijnstate Hospital, Arnhem, the Netherlands; 3 Department of Psychiatry, UMC Groningen, University of Groningen, Groningen, the Netherlands; 4 Donders Center, Nijmegen, the Netherlands; 5 Department of Psychiatry, Radboud UMC, Nijmegen, the Netherlands; 6 Amsterdam Brain & Cognition, University of Amsterdam, Amsterdam, the Netherlands

## Abstract

**Importance:**

Although electroconvulsive therapy (ECT) is often effective, approximately half of patients with depression undergoing ECT do not benefit sufficiently, and relapse rates are high. ECT sessions have been shown to weaken reactivated memories. The effect of emotional memory retrieval on cognitive schemas remains unknown.

**Objective:**

To assess whether emotional memory retrieval just before patients receive ECT sessions weakens underlying cognitive schemas, improves ECT effectiveness, increases ECT response, and reduces relapse rates.

**Design, Setting, and Participants:**

In this multicenter randomized clinical trial conducted from 2014 to 2018 in the departments of psychiatry in 3 hospitals in the Netherlands, 72 participants were randomized 1:1 to 2 parallel groups to receive either emotional memory reactivation (EMR-ECT) or control memory reactivation (CMR-ECT) interventions before ECT sessions. The Hamilton Depression Rating Scale (HDRS [total score range: 0-52, with 0-7 indicating no depression and ≥24 indicating severe depression]) was used to measure symptoms of depression during and after ECT, with a 6-month follow-up period. Participants were between ages 18 and 70 years with a primary diagnosis of unipolar major depressive disorder (MDD) according to the *Diagnostic and Statistical Manual of Mental Disorders* (Fourth Edition, Text Revision) and in whom ECT was indicated. Data analysis was performed from July to November 2019.

**Interventions:**

EMR-ECT or CMR-ECT interventions prior to ECT sessions.

**Main Outcomes and Measures:**

Depression scores and relapse rates within 6 months were assessed with the HDRS and analyzed using logistic and linear multiple regression analyses.

**Results:**

A total of 66 patients (mean [SD] age, 49.3 [12.3] years; 39 [59.1%] women) were randomized to the EMR-ECT group (n = 32) or the CMR-ECT group (n = 34). Regardless of the memory intervention, 42.4% (28 of 66) of patients responded (≥50% decrease of symptom severity on the HDRS). Of patients who responded, 39.3% (11 of 28) relapsed within 6 months. Remission rates (CMR-ECT group, 29.4% [10 of 34] vs EMR-ECT group, 25.0% [8 of 32]; *P* = .58), mean (SD) HDRS scores after the ECT course (CMR-ECT group, 14.6 [8.6] vs EMR-ECT group, 14.9 [8.8]; *P* = .88), total mean (SD) number of required ECT sessions for response (CMR-ECT group, 14.9 [7.9] vs EMR-ECT group, 15.6 [7.3]; *P* = .39), and relapse rates (CMR-ECT group, 46.7% [7 of 15] vs EMR-ECT group, 30.8% [4 of 13]; *P* = .33) were not significantly altered by the intervention.

**Conclusions and Relevance:**

Study findings suggest that the EMR-ECT intervention just before patient receipt of ECT for depression did not improve effectiveness, increase speed of response, or reduce relapse rates after the ECT course compared with patients receiving CMR-ECT.

**Trial Registration:**

Trialregister.nl Identifier: NL4289

## Key Points

**Question:**

Can emotional memory retrieval just prior to electroconvulsive therapy (ECT) sessions improve the outcome of ECT in patients with major depressive disorder?

**Findings:**

In this randomized clinical trial, 66 patients received emotional memory reactivation or a control condition prior to ECT. The intervention did not influence remission rates, depression scores after the ECT course, total completed ECT sessions, or relapse rates.

**Meaning:**

Personalized reactivation of emotional memories just prior to ECT sessions did not improve ECT effectiveness or speed of response and did not reduce relapse rates.

## Teaser

This randomized clinical trial assesses whether emotional memory retrieval in adult patients before receiving electroconvulsive therapy (ECT) weakens underlying cognitive schemas, improves ECT effectiveness, increases ECT response, and reduces relapse rates.

## Introduction

Major depressive disorder (MDD) is a common mental disorder associated with substantial reductions in daily functioning. Initial treatment for MDD consists of psychotherapy and/or pharmacotherapy. A 2010 study^1^ reported that, even after receiving 4 different pharmacotherapeutic interventions, more than half of patients with depression do not recover. Electroconvulsive therapy (ECT) has been reported to be beneficial in patients with MDD resistant to pharmacologic treatment,^2^ although half of the patients undergoing ECT will not achieve full remission.^3^ Moreover, relapse rates after successful ECT are high, as one-third of patients can be expected to relapse within 6 months.^4^ More effective targeting of specific underlying psychopathological mechanisms of MDD may help improve ECT effectiveness, be associated with more rapid ECT response times, and decrease relapse rates after successful ECT.

Cognitive schemas are relatively stable thought representations of prior knowledge and experiences. Cognitive theory holds that activated negative schemas play an important etiologic role in MDD, as activated negative schemas may be factors in information processing.^5^ Weakening of negative schemas and change from maladaptive to more adaptive schema processing have been hypothesized to underlie recovery of patients from MDD, but weakening may also lower the relapse rate after cognitive behavioral therapy.^5,6^ Cognitive schemas are embedded in strong associative memory structures.^7^

Research indicates that when memories are reactivated they may become temporarily labile and require restabilization processes to be maintained, a process known as reconsolidation. Pharmacologic interventions that disrupt the restabilization processes may selectively weaken the reactivated memory.^8^ Studies in the 1960s and 1970s reported that electroconvulsive treatment disrupted reactivated memories in rats^9^ and that reactivation of obsessive-compulsive symptoms in patients before applying ECT increased effectiveness.^10^ In a 2014 study,^11^ a single ECT session selectively impaired memory for a learned emotional story when reactivated just prior to an ECT session. If a single ECT session could weaken memory, multiple emotional memory reactivations (EMRs) in consecutive ECT sessions may improve ECT effectiveness, be associated with more rapid ECT response times, and reduce relapse rates after successful ECT.

In this randomized clinical trial (RCT), patients with MDD were randomized to receive either an autobiographical EMR or a control memory reactivation (CMR) not associated with the patients’ depression just before each ECT session (EMR-ECT and CMR-ECT, respectively). We hypothesized that reactivation of patients’ own emotional memories related to MDD just before receipt of ECT sessions may weaken their associated negative cognitive schemas, resulting in (1) higher remission rates and lower depression severity scores after the ECT course, (2) fewer required ECT sessions to reach a response, and (3) lower relapse rates within 6 months of the ECT course.

## Methods

### Study Sites and Participants

Patients in this multicenter study were recruited from the department of psychiatry from 3 hospitals in the Netherlands (Rijnstate Hospital, Arnhem; Amsterdam University Medical Center, Amsterdam; and University Medical Center Groningen, Groningen) from 2014 to 2018. Eligible participants were patients aged between 18 and 70 years primarily diagnosed with unipolar MDD, fulfilling all criteria of the *Diagnostic and Statistical Manual of Mental Disorders* (Fourth Edition, Text Revision)^12^ (*DSM-IV-TR*), for whom ECT was indicated. All patients had a history of insufficient response to previous treatments (pharmacotherapy and psychotherapeutic interventions), which is the primary indication for ECT in the Netherlands.^13^ Patients were willing and able to understand, to participate, and to comply with the study requirements. Exclusion criteria were the presence of psychotic features (owing to the possibility that psychotic features might worsen because of the cognitive intervention itself); bipolar disorder; schizophrenia or other primarily psychotic disorders; substance abuse; and other cognitive disorders. Psychiatric diagnoses were classified according to *DSM-IV-TR* criteria using the Mini-International Neuropsychiatric Interview.^14^ Participants received compensation (€40) when they completed the study. The study protocol was approved by the Medical Ethical Committee of the Amsterdam University Medical Center and registered in the Dutch Trial Register (NL4289). All patients provided written informed consent, and all procedures were carried out in accordance with the tenets of the Declaration of Helsinki.^15^ This study followed the Consolidated Standards of Reporting Trials (CONSORT) reporting guideline for RCTs. The trial protocol is available in Supplement 1.

### Randomization and Design

Participants were randomly assigned 1:1 to 2 parallel groups, EMR-ECT or CMR-ECT, by means of a predefined randomization list, stratified for each treatment center, using blocks of 4 to ensure equal group sizes. Concealment of randomization was maintained by access to randomization lists only by study investigators who were not directly treating or assessing eligible patients. The ECT teams and clinical outcome assessors were all blinded to randomization.

Power calculation indicated a total sample size of 98 patients to detect a medium effect size (25% higher remission rate, assuming a 42% base rate in a previous trial^13^) with 80% power at 1-tailed α of .05. The RCT continued after an interim analysis with 38 patients that was able to detect a statistical trend (*P* = .10) with 80% power. Critical *z* values with O’Brien-Fleming correction were 3.11 for the interim analysis and 1.97 for the final analysis. The RCT was terminated prematurely after including 72 patients, as inclusion decreased substantially because of a change in the Dutch mental health care policy. The power to detect the same effect size (25% higher remission rate) had decreased to 69%, but the RCT still had 80% power to detect a 29% higher remission rate.

### Intervention

In patients receiving EMR-ECT treatment, autobiographical memories associated with maladaptive schemas were identified and reactivated according to a standardized protocol. First, an experienced psychologist, in collaboration with the patient, determined which memories to activate. Recurring maladaptive schema thoughts were identified using the Automatic Thoughts Questionnaire-Revised.^16^ Subsequently, patients selected 6 maladaptive thoughts most central to their current depressive episode. These 6 thoughts were narrowed to 3 by determining which thoughts were most closely related to autobiographical episodes. These autobiographical episodes were then written down in short narratives as vividly as possible (ie, events written as detailed as possible including feelings, thoughts, sensory modalities, and involvement of other people).

During the ECT course, the assigned memory reactivation intervention was applied in the waiting room where the patients were prepared for ECT. In the EMR-ECT group at approximately 10 minutes before application of the ECT stimulus, a research assistant reactivated the autobiographical episode by reading 1 of the narratives slowly and carefully, providing the patient time to recall memory in detail and lasting approximately 3 minutes. Only 1 autobiographical memory was reactivated per ECT session, alternating between the 3 selected narratives.

In the CMR-ECT group, an identical procedure was followed. Instead of autobiographical EMR, the research assistant applied a 3-minute control memory intervention that was related to the importance of sleep, physical exercise, and substance use in mental health. After that process, patients received ECT sessions according to Dutch national ECT guidelines.^13,17^

### Psychometric Instruments

Patients were evaluated before the start of the ECT course; after 6, 12, and 18 ECT sessions; and within 2 weeks after the last ECT session. Follow-up evaluations were done at 1, 2, 4, and 6 months. At each evaluation, the Hamilton Depression Rating Scale^18^ (HDRS [total score range: 0-52, with 0-7 indicating no depression and ≥24 indicating severe depression]) was used to measure depressive symptomatology by trained research nurses blinded for treatment randomization.^19^ The HDRS is a valid observer-rated instrument consisting of 17 items with a maximum score of 52 (mean weighted sum score interrater coefficient: κ = 0.92).^20^ Remission was defined as an HDRS score less than or equal to 7, response as a 50% or more reduction in HDRS score after the ECT course compared with baseline,^21^ and relapse as an increase of 10 or more HDRS points on at least 1 assessment in the 1- to 6-month follow-up period.

To quantify treatment resistance at baseline, we sued the Dutch Measure for Quantification of Treatment Resistance in Depression (DM-TRD), which consists of 11 items with a maximum score of 27 and has good psychometric properties and predictive validity.^22^ In addition, the Dutch version of the national adult reading test was used as a proxy for IQ.

### Electroconvulsive Therapy

After intravenous induction of anesthesia with etomidate (0.2 mg/kg body mass), muscle paralysis with succinylcholine (0.5-1 mg/kg body mass), and application of appropriate oxygenation (100% oxygen, positive pressure) until the resumption of spontaneous respiration, ECT was administered using a constant-current (0.9 A), brief-pulse (0.5 milliseconds) device (Thymatron IV; Somatics Incorporation). Lithium was tapered before starting the ECT course; other concomitant medications were kept constant. ECT sessions were performed twice a week. Patients started with 6 right unilateral (RUL) ECT sessions unless clinicians decided to start with bifrontotemporal (BL) electrode placement because of severe clinical conditions or previous effective BL-ECT. During the first session, the seizure threshold was estimated by an internationally accepted, empirical, age-adjusted titration method, and the personalized dose was estimated as 6 or 2.5 times standard treatment for RUL or BL-ECT, respectively.^13^ ECT courses were discontinued when remission was achieved (HDRS score ≤7) or when no further improvement was observed over a period of 2 weeks. The total number of administered ECT sessions was registered for each patient.

### Statistical Analysis

Baseline characteristics between both groups were analyzed using 2-sample *t* tests, χ^2^ tests, or Mann-Whitney tests as appropriate. For the whole study group, differences in HDRS scores before and after the ECT course were analyzed using a paired *t* test, and response and remission rates were calculated as percentages.

To investigate the relapse rates within 6 months, logistic and linear multiple regression analyses were used, with remission status (logistic) or HDRS score after the ECT course (linear) used as the dependent variable, and the intervention (EMR-ECT or CMR-ECT) as the predictor variable, with sex, age, baseline HDRS score, final electrode placement, and treatment site as covariates. This approach was different from the original analysis plan that consisted of testing only remission rates.

To explore the secondary outcomes (number of required ECT sessions to reach response and relapse rate), linear (number of ECT sessions) and logistic (relapse rate) multiple regression analyses were conducted in the patients showing response to ECT (n = 28). The number of required ECT sessions was square root transformed to reduce skewness. Because time to relapse was not recorded accurately for the intended survival analysis, we analyzed the relapse rate. In the models, the intervention was entered as the predictor variable, and sex, age, HDRS score before (number of ECT sessions) or after (relapse rate) the ECT course, final electrode placement, and treatment site were entered as covariates. All analyses were conducted using SPSS statistical software, version 25 (IBM Corp), and *P* < .05 denoted statistical significance. Data analysis was performed from July to November 2019.

## Results

**Figure 1.**
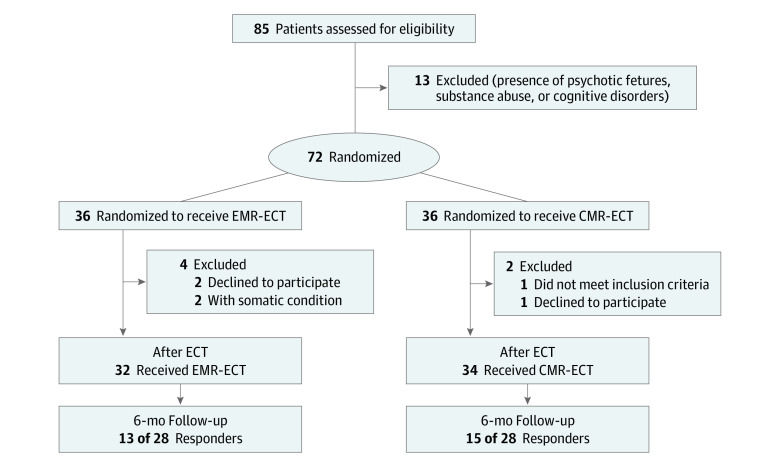
Patient Flow Diagram CMR-ECT indicates control memory reactivation ECT; ECT, electroconvulsive therapy; and EMR-ECT, emotional memory reactivation ECT.

During the study period, 72 patients were randomized. Six patients dropped out: 3 patients with anxiety about the ECT session requested not to be contacted further just before the ECT sessions, 2 patients discontinued ECT because of non–ECT related medical conditions, and 1 patient disclosed severe benzodiazepine addiction. Therefore, 66 patients (mean [SD] age, 49.3 [12.3] years; 39 [59.1%] women) were included in the final analyses (Figure 1). No differences appeared in age, sex, IQ, DM-TRD, and HDRS score at baseline between patients receiving EMR-ECT (n = 32) and patients receiving CMR-ECT (n = 34) (Table 1). Regardless of the memory intervention, the mean post-ECT HDRS score improved significantly (*t*_65_ = 8.1; *P* < .01; Cohen *d_z_* = 1.00). Twenty-eight of 66 patients (42.4%) showed response and 18 of 66 (27.3%) remission, which was lower than expected^13^ (Table 2).

**Table 1.** Patient Characteristics

Patient characteristic	Mean (SD)			*P* value
	Total sample	EMR-ECT	CMR-ECT	
No. (%)	66 (100)	32 (48)	34 (52)	
Age	49.3 (12.3)	49.6 (11.4)	48.9 (13.2)	.78^a^
Female sex, No. (%)	39 (59.1)	20 (62.5)	19 (55.9)	.59^b^
IQ	99.7 (15.9)	98.7 (14.4)	100.8 (16.6)	.62^a^
DM-TRD	15.0 (2.8)	15.1 (3.0)	14.8 (2.7)	.65^a^
HDRS score at baseline	24.9 (6.0)	26.0 (5.9)	23.9 (5.9)	.15^a^

Abbreviations: CMR-ECT, control memory reactivation ECT; DM-TRD, Dutch Measure for quantification of Treatment in Depression; ECT, electroconvulsive therapy; EMR-ECT, emotional memory reactivation ECT; HDRS, Hamilton Depression Rating Scale (total score range: 0-52, with 0-7 indicating no depression and ≥24 indicating severe depression).

^a^ Two-sample *t* test.

^b^ χ^2^ Test.

**Table 2.** Outcome and Treatment Characteristics for EMR-ECT and CMR-ECT

Variable	No. (%)			*P* value^a^
	Total sample	EMR-ECT	CMR-ECT	
No. (%)	66 (100)	32 (48)	34 (52)	
Outcome				
HDRS score after ECT course, mean (SD)	14.8 (8.6)	14.9 (8.8)	14.6 (8.6)	.89^b^
Response rate (N = 66)^c^	28 (42.4)	13 (40.6)	15 (44.1)	.77^d^
Remission rate (N = 66)	18 (27.3)	8 (25.0)	10 (29.4)	.69^d^
Relapse rate (N = 28)	11 (39.3)	4 (30.8)	7 (46.7)	.39^d^
Dropout rate from study (N = 72)	6 (8.3)	4 (11.1)	2 (5.5)	.39^d^
Treatment characteristic				
Final electrode placing is RUL	42 (63.6)	19 (59.4)	23 (67.6)	.49^d^
Total necessary ECT sessions during the course, mean (SD)	15.2 (7.5)	15.6 (7.3)	14.9 (7.9)	.71^e^

Abbreviations: CMR-ECT, control memory reactivation ECT; ECT, electroconvulsive therapy; EMR-ECT, emotional memory reactivation ECT; HDRS, Hamilton Depression Rating Scale (total score range: 0-52, with 0-7 indicating no depression and ≥24 indicating severe depression); RUL, right unilateral ECT.

^a^ The *P* values are not corrected for covariates and therefore differ from the *P* values from the multiple regression analyses in the results.

^b^ Two-sample *t* test.

^c^ Responders to ECT showed 50% or more decrease of symptom severity on the HDRS, and remitters showed an HDRS score 7 or less after the ECT course.

^d^ χ^2^ Test.

^e^ Mann-Whitney test.

**Figure 2.**
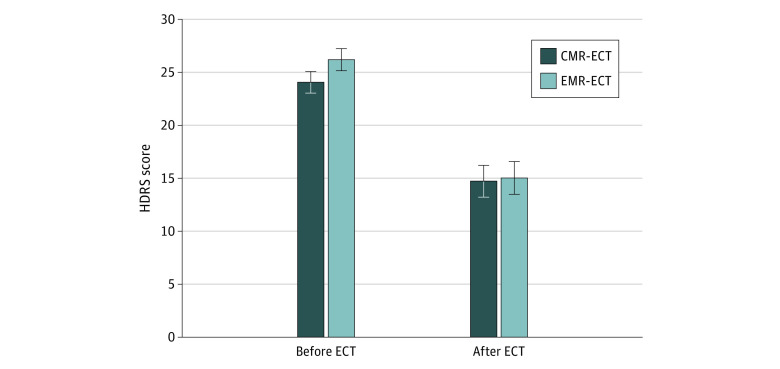
Hamilton Depression Rating Scale (HDRS) Score Before and After Electroconvulsive Therapy (ECT) Data are expressed as mean (SEM). CMR-ECT indicates control memory reactivation ECT; and EMR-ECT, emotional memory reactivation ECT.The HDRS has a total score range of 0 to 52, with 0 to 7 indicating no depression and 24 or greater indicating severe depression.

Logistic regression analysis showed no significant effect of the memory intervention on remission rate (CMR-ECT group, 29.4% [10 of 34] vs EMR-ECT group, 25.0% [8 of 32]; β = 0.33; *P* = .58; odds ratio, 1.39). Linear regression analysis showed no significant effect of the memory intervention on post-ECT HDRS scores (CMR-ECT group, 14.6 [8.6] vs EMR-ECT group, 14.9 [8.8]; β = 0.02; *P* = .88; semipartial *r*^2^ < 0.01) (Figure 2).

Linear regression analysis showed no significant association between the memory intervention and the required total amount of ECT sessions to reach response (n = 28; CMR-ECT group, 14.9 [7.9] vs EMR-ECT group, 15.6 [7.3]; β = 0.14; *P* = .39; semipartial *r*^2^ = 0.02), although the covariate final electrode placement (β = 0.41; *P* = .02; semipartial *r*^2^ = 0.12) was significant. As expected, patients treated with BL required more ECT sessions than patients treated only with RUL, as most patients receiving BL initially received RUL-ECT as well.

Twenty-eight patients showed response and 11 of those (39.3%) relapsed within 6 months. In ECT responders in the separate intervention groups, 4 of 13 patients (30.8%) receiving EMR-ECT relapsed and 7 of 15 patients (46.7%) receiving CMR-ECT relapsed. Logistic regression analysis showed no significant effect of the intervention on relapse rates within 6 months (β = 0.93; *P* = .33; odds ratio = 2.53).

## Discussion

In this RCT, reactivation of personalized emotional memories just prior to ECT sessions showed no better ECT outcome than a control intervention. The memory intervention neither increased the rate of the ECT response nor reduced the relapse rate within the 6-month follow-up. We aimed to translate laboratory research into our clinical MDD treatment program.^23^ Therefore, as the EMR intervention was based on reconsolidation theory and only 1 ECT session already showed weakened memories in a previous clinical study,^11^ our findings were not as expected. However, several lessons may be learned from this RCT that, to our knowledge, was the first of its kind.

We attempted to weaken emotional memories with regular ECT sessions by disrupting the reconsolidation process of reactivated personalized emotional cognitive schemas. This attempt may have failed because older emotional memories were too resistant to change. Cognitive schemas underlying MDD are expected to be formed in the adolescent period. Therefore, the age and strength of these memories may have reduced the ability of our reactivation cues to induce destabilization of the very old underlying memories.^24,25^ Furthermore, memories are redundantly encoded, which means that a minor disturbance does not impair its representation. However, a study from 1976 suggested that acting out obsessive-compulsive symptoms just before unmodified ECT sessions might help improve results for patients.^10^ Administration of ECT without anesthesia is now considered unethical. But based on these results^10^ and insights from reconsolidation theory,^11^ we considered an EMR intervention in patients undergoing ECT to be worth investigating, as new approaches are needed to further improve ECT efficacy and prevent relapse.

The type of memory that was reactivated may not have been sensitive to modulation by our chosen memory intervention. Most earlier studies on the reconsolidation theory examined pavlovian responses, but only a few studied the effect of disruption of episodic memory.^11^ In our study, even forms of semantic memory were targeted, which may possibly be more rigid to modulation. Further studies are needed to examine which types of memory have utility in treatment of MDD and can be modulated.

In addition to the type of reactivated memory, the reactivation cues used in our study may not have been suitable to reactivate the underlying emotional cognitive schemas. The reactions of most patients, however, indicated that the personalized emotional memory script triggered strong emotions. Theoretically, these strong emotions could trigger negative schemas, but it was not possible to ascertain whether this mechanism actually occurred in the patients receiving EMR-ECT. Included patients had difficulty in identifying their negative schemas (eg, they overgeneralized their negative memories). Therefore, it was decided to use detailed memories, including negative emotions and cognitions, instead of more abstract underlying negative cognitive schemas. It is possible that limited destabilization of such intended abstract schemas decreased the effect of our memory intervention.

A 2017 study^26^ showed that new learning might be necessary for reconsolidation to occur. As we reactivated only old memories and did not enforce new learning, destabilization of bad memories was not provoked. In future studies, other personalized cues may be invented in which the aspect of new learning for patients is taken into account. At such time, reactivation of underlying negative schemas just before an ECT session may be more beneficial.

The procedure of administering the reminders may have affected our results. In line with a rodent study, our memory reactivation paradigm was 3 to 5 minutes.^27^ Other studies, however, suggest that this duration may have been too long.^11,24,28,29^ Conversely, this duration may have been too short, as a reminder duration of 10 to 30 minutes was recently found to be effective for posttraumatic stress disorder.^30^ In addition, the reactivation procedure took place approximately 10 minutes before the actual ECT session so as to blind the treating physicians and to perform ECT according to regular practice. However, this delay may have been too long, as Kroes et al^11^ reactivated the memory within a few minutes before induction of anesthesia.

In this study, the memory intervention was well tolerated by patients, and the overall dropout rate was low, suggesting similar interventions are feasible. Given the possible lessons of our RCT, future studies may consider (1) reactivation of more recent memories; (2) creation of more appropriate reminders (eg, newly learned regarding the episodic memory); (3) use of reminders of a different duration; and (4) use of a shorter duration between reactivation of the reminder and the ECT stimulus (eg, 1-5 minutes before induction of anesthesia).

### Limitations

This study has limitations. From a methods standpoint, an important problem was the inability of verifying reactivation of negative memories or schemas in patients. However, our research assistants noticed emotional reactions in patients when listening to their personal negative experiences, which may indicate reactivation of negative memory. Furthermore, the control group received potentially useful psychoeducation that was necessary to maintain patient blinding but that might also have contributed to the antidepressant effects in this group and the null findings. Converesely, this psychoeducation might have been forgotten as well because of the intervention, reducing its antidepressant effects. This possible confounder of an antidepressant effect in the control condition usually affects clinical trials with psychological interventions.

In this RCT, the response rate was lower than expected, limiting the power to detect differences in relapse rates after ECT response; the power to detect differences in relapse rates was reduced at the outset, as this analysis was restricted to treatment responders. Highly selected groups of patients with treatment-resistant MDD may show ECT remission rates of 48%,^3^ whereas our 27.3% remission rate was consistent with that of a community sample.^31^ Moreover, our DM-TRD scores appeared to be much higher than others in treatment-resistant MDD groups,^22^ and we excluded patients with a higher chance of successful ECT (ie, age >70 years; psychotic depression), which may have contributed to the low remission rate. Conversely, the low response rate could have maximized the probability to show efficacy of the EMR-ECT.

## Conclusions

In this study, personalized reactivation of emotional memories just before ECT sessions for MDD was well tolerated but did not improve ECT efficacy, decrease the time to response, or reduce the relapse rate. This RCT highlights the difficulties of translating insights from laboratory research into clinical practice and may provide direction for future studies to further improve ECT for patients with severe MDD.

## Notes

Supplement 1. **Trial Protocol**

Supplement 2. **Data Sharing Statement**
