# Supplementary material for: Effectiveness of Emotional Memory Reactivation vs Control Memory Reactivation Before Electroconvulsive Therapy in Adult Patients With Depressive Disorder: A Randomized Clinical Trial
Source: JAMA Netw Open. 2020 Aug 4;3(8):e2012389. doi: 10.1001/jamanetworkopen.2020.12389 (PMC7403919; doi:10.1001/jamanetworkopen.2020.12389)
Supplement: Supplement 1. — Trial Protocol [file jamanetwopen-3-e2012389-s001.pdf]

SCHEMA-ECT: A NOVEL TREATMENT FOR SEVERE DEPRESSION

November 3, 2015

METc 2013/351

SCHEMA-ECT: A NOVEL TREATMENT FOR SEVERE DEPRESSION
(“EEN GECOMBINEERDE ECT EN GESPREKSBEHANDELING VOOR DEPRESSIE”)

**Protocol ID**	**N/A**
**Short title**	**Schema-ECT**
**EudraCT number**	**N/A**
**Version**	**2**
**Date**	**November 3, 2015**
**Coordinating investigator/project leader**	Dr. G.A. van Wingen<br>Department of Psychiatry<br>Academic Medical Center<br>Amsterdam
**Principal investigator(s) (in Dutch: hoofdonderzoeker/ uitvoerder)**<br>***<Multicenter research: per site>***	**Academic Medical Center**<br>Dr. G.A. van Wingen<br>Department of Psychiatry<br>Academic Medical Center; PO Box 22660<br>1100 DD Amsterdam<br>E-mail: g.a.vanwingen@amc.nl<br>Tel: 020-89 13523<br><br>Drs. D.S. Scheepens<br>Department of Psychiatry<br>Academic Medical Center; PO Box 22660<br>1100 DD Amsterdam<br>E-mail: d.s.scheepens@amc.uva.nl<br>Tel: 020-89 13638<br><br>**University Medical Center Groningen**<br>Dr. H.G. Ruhe<br>Mood and Anxiety Disorders UCP, UMCG<br>Hanzeplein 1<br>9700 RB Groningen<br>E-mail: H.G.Ruhe@UMCG.NL / H.G.Ruhe@gmail.com<br>Tel: 050-3612367

	**Rijnstate Hospital**<br>Dr. J.A. van Waarde<br>Rijnstate Hospital<br>P.O. box 9555<br>6815 AD Arnhem<br>E-mail: jvanwaarde@rijnstate.nl<br>Tel: 088-005 8934
**Sponsor (in Dutch: verrichter/opdrachtgever)**	**AMC**
**Subsidising party**	**Hersenstichting**
**Independent expert (s)**	**Academic Medical Center**<br>Dr. J.G. Storosum<br>Department of Psychiatry<br>Academic Medical Center, PO Box 22660<br>1100 DD Amsterdam<br>E-mail: j.g.storosum@amc.uva.nl<br><br>**University Medical Center Groningen**<br>Drs. W. Veling<br>University Medical Center Groningen<br>Hanzeplein 1<br>9700 RB Groningen<br>E-mail: w.veling@umcg.nl<br><br>**Rijnstate Hospital**<br>Dr. B Verwey<br>P.O. box 9555

	6815 AD Arnhem<br><br>E-mail: basverwey@gmail.com
**Laboratory sites <*if applicable*>**	**N/A**
**Pharmacy <*if applicable*>**	**N/A**

PROTOCOL SIGNATURE SHEET

**Name**	**Signature**	**Date**
**Sponsor or legal representative:**<br>**Head of Department:**<br>***Prof. dr. D.A.J.P. Denys***	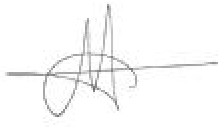	**November 3, 2015**
**[Coordinating Investigator/Project leader/Principal Investigator]:**<br>***Dr. G.A. van Wingen***	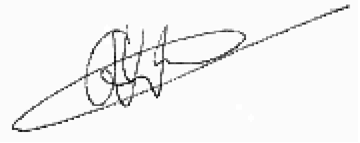	**November 3, 2015**

TABLE OF CONTENTS

1. INTRODUCTION AND RATIONALE 11
2. OBJECTIVES 15
3. STUDY DESIGN 16
4. STUDY POPULATION 17
4.1 Population (base) 17
4.2 Inclusion criteria 17
4.3 Exclusion criteria 17
4.4 Sample size calculation 17
5. TREATMENT OF SUBJECTS 18
5.1 Investigational product/treatment 18
5.2 Use of co-intervention (if applicable) 18
5.3 Escape medication (if applicable) 18
6. INVESTIGATIONAL PRODUCT 19
6.1 Name and description of investigational product(s) 19
6.2 Summary of findings from non-clinical studies 19
6.3 Summary of findings from clinical studies 19
6.4 Summary of known and potential risks and benefits 19
6.5 Description and justification of route of administration and dosage 19
6.6 Dosages, dosage modifications and method of administration 19
6.7 Preparation and labelling of Investigational Medicinal Product 19
6.8 Drug accountability 19
7. NON-INVESTIGATIONAL PRODUCT 20
7.1 Name and description of non-investigational product(s) 20
7.2 Summary of findings from non-clinical studies 20
7.3 Summary of findings from clinical studies 20
7.4 Summary of known and potential risks and benefits 20
7.5 Description and justification of route of administration and dosage 20
7.6 Dosages, dosage modifications and method of administration 20
7.7 Preparation and labelling of Non Investigational Medicinal Product 20
7.8 Drug accountability 20
8. METHODS 21
8.1 Study parameters/endpoints 21
8.1.1 Main study parameter/endpoint 21
8.1.2 Secondary study parameters/endpoints (if applicable) 21
8.1.3 Other study parameters (if applicable) 21
8.2 Randomisation, blinding and treatment allocation 22
8.3 Study procedures 22
8.4 Withdrawal of individual subjects 24
8.4.1 Specific criteria for withdrawal (if applicable) 25
8.5 Replacement of individual subjects after withdrawal 25
8.6 Follow-up of subjects withdrawn from treatment 25

8.7 Premature termination of the study 25
9. SAFETY REPORTING 25
9.1 Temporary halt for reasons of subject safety 25
9.2 AEs, SAEs and SUSARs 25
9.2.1 Adverse events (AEs) 25
9.2.2 Serious adverse events (SAEs) 26
9.2.3 Suspected unexpected serious adverse reactions (SUSARs) 26
9.3 Annual safety report 26
9.4 Follow-up of adverse events 27
9.5 [Data Safety Monitoring Board (DSMB) / Safety Committee] 27
10. STATISTICAL ANALYSIS 28
10.1 Primary study parameter(s) 28
10.2 Secondary study parameter(s) 28
10.3 Other study parameters 28
10.4 Interim analysis (if applicable) 28
11. ETHICAL CONSIDERATIONS 29
11.1 Regulation statement 29
11.2 Recruitment and consent 29
11.3 Objection by minors or incapacitated subjects (if applicable) 29
11.4 Benefits and risks assessment, group relatedness 29
11.5 Compensation for injury 30
11.6 Incentives (if applicable) 30
12. ADMINISTRATIVE ASPECTS, MONITORING AND PUBLICATION 31
12.1 Handling and storage of data and documents 31
12.2 Monitoring and Quality Assurance 31
12.3 Amendments 31
12.4 Annual progress report 31
12.5 End of study report 31
12.6 Public disclosure and publication policy 32
13. STRUCTURED RISK ANALYSIS 33
13.1 Potential issues of concern 33
13.2 Synthesis 33
14. REFERENCES 34

LIST OF ABBREVIATIONS AND RELEVANT DEFINITIONS

**ABR**	**ABR form, General Assessment and Registration form, is the application form that is required for submission to the accredited Ethics Committee (In Dutch, ABR = Algemene Beoordeling en Registratie)**
**AE**	**Adverse Event**
**AR**	**Adverse Reaction**
**CA**	**Competent Authority**
**CCMO**	**Central Committee on Research Involving Human Subjects; in Dutch: Centrale Commissie Mensgebonden Onderzoek**
**CV**	**Curriculum Vitae**
**DSMB**	**Data Safety Monitoring Board**
**EU**	**European Union**
**ECS**	**Electroconvulsive Shock therapy (ECT in animal models)**
**ECT**	**Electroconvulsive Therapy**
**EudraCT**	**European drug regulatory affairs Clinical Trials**
**GCP**	**Good Clinical Practice**
**IB**	**Investigator's Brochure**
**IC**	**Informed Consent**
**IMP**	**Investigational Medicinal Product**
**IMPD**	**Investigational Medicinal Product Dossier**
**METC**	**Medical research ethics committee (MREC); in Dutch: medisch ethische toetsing commissie (METC)**
**MRI**	**Magnetic Resonance Imaging**
**(S)AE**	**(Serious) Adverse Event**
**SPC**	**Summary of Product Characteristics (in Dutch: officiële productinfomatie IB1-tekst)**
**Sponsor**	**The sponsor is the party that commissions the organisation or performance of the research, for example a pharmaceutical company, academic hospital, scientific organisation or investigator. A party that provides funding for a study but does not commission it is not regarded as the sponsor, but referred to as a subsidising party.**
**SUSAR**	**Suspected Unexpected Serious Adverse Reaction**
**Wbp**	**Personal Data Protection Act (in Dutch: Wet Bescherming Persoonsgevens)**
**WMO**	**Medical Research Involving Human Subjects Act (in Dutch: Wet Medisch-wetenschappelijk Onderzoek met Mensen**

SUMMARY

Rationale: Electroconvulsive therapy (ECT) is often considered a last treatment option for otherwise treatment resistant depression. Unfortunately, approximately 50% of patients do not respond sufficiently (Heijnen et al., 2010). Furthermore, of the patients who respond initially, 40-80% relapse within half a year (Sackeim et al., 2001). We hypothesize that suboptimal efficacy of ECT could be due to insufficient modulation of negative cognitive schemas, which are relative stable representations of prior knowledge and experiences. These negative schemas distort the perception of new experiences in a maladaptive manner, and focus one's thoughts on negative aspects of oneself. Cognitive theories of depression hold that these negative schemas play an important role in the development, maintenance and recurrence of depression (Beck and Clark, 1988).
We recently found that memories can be weakened by applying ECT shortly after reactivation of a memory (Kroes et al., 2013). This suggests that reactivation of negative schemas just prior to ECT may also weaken those schemas. According to the cognitive theory of depression this will lead to the recovery from depression and will additionally reduce the risk to relapse, but this has not yet been investigated. Here, we aim to investigate the efficacy of "schema-ECT" and hypothesize that repeated reactivation of depressive schemas prior to ECT weakens negative schemas, increases the efficacy of the ECT course, and reduces the relapse rate after the reduction of the ECT session frequency or discontinuing ECT. In addition to our main aim, we will investigate whether the clinical response to ECT response is associated with changes in neurobiological biomarkers using neuroimaging scans and blood samples obtained from the venous catheter, and whether these biomarkers at baseline can predict treatment response (van Waarde et al., submitted).

Objective: Our primary objectives are to determine whether schema-ECT increases the remission rate of a course of ECT, reduces the relapse rate, and weakens negative schemas. Our secondary objectives are to identify neurobiological biomarkers that predict and are associated with treatment response.

Study design: A randomized controlled trial (RCT) is used to determine schema-ECT efficacy. Neuroimaging and blood biomarkers will be associated with changes in clinical variables.

Study population: 98 patients with a primary diagnosis of major depressive disorder (MDD) and an indication for ECT between 18-70 years of age. Eligible candidates for MRI will be asked to participate in the neuroimaging experiment.

Intervention (if applicable): Patients will be randomized to schema-ECT or control-ECT, stratified for research center. Schema-ECT consists of reactivation of depressive schemas using the arrow-down technique that is used in cognitive-behavioral therapy (CBT). In the

control condition, patients will be interviewed about details that are also of clinical relevance but are not expected to activate depressive schemas (e.g., their medical record, diet, exercise). ECT is performed according to the national guidelines, which consists of a minimum of 6 biweekly sessions until remission or a plateau in response is achieved.

Main study parameters/endpoints: Treatment efficacy as measured with the Hamilton Rating Scale for Depression (HAM-D; 17-items). Response is defined as a 50% reduction and remission as a score ≤7. The influence on negative schemas is measured with the Dysfunctional Attitude Scale (DAS), the Automatic Thoughts Questionnaire (ATQ), and the Self-Referent Encoding Task (SRET). Neuroimaging biomarkers are hippocampal magnetic resonance spectroscopy (MRS), diffusion tensor imaging (DTI), and resting-state functional magnetic resonance imaging (fMRI). Blood biomarkers will be determined from blood samples obtained from the venous catheter that is placed for anaesthesia induction during the treatment phase or venapuncture at follow-up.

Nature and extent of the burden and risks associated with participation, benefit and group relatedness: The burden of ineffectively treated depression is high. The burden of ECT is considerable but is warranted because of successful treatment, and its risk can be considered negligible. Importantly, only the regular ECT-population will be recruited. The additional burden for participating in this study is minimal and the additional risk can be considered negligible. Because the treatment under investigation is expected to increase the efficacy of ECT, patients may directly benefit from participating in this study. The additional burden for participating in the neuroimaging study can be considered minimal, and the additional risk for eligible candidates is negligible. The additional burden for blood sampling from the venous catheter that is already placed as part of the ECT procedure can be considered minimal and the risk negligible.

1. INTRODUCTION AND RATIONALE

Severe depression

Major depressive disorder (MDD) is a common psychiatric disorder that is associated with significant functional impairment. Many depressed patients can be treated successfully with antidepressants or psychotherapy. However, the largest clinical trial in the United States showed that at least one third of the patients do not recover even after four successive steps of pharmacological/psychotherapeutic treatment, and that 21% of depressed patients meet the criteria for chronic depression (duration longer than 2 years) (Rush et al., 2006). Electroconvulsive therapy (ECT) is an important treatment option for such severe and otherwise treatment resistant patients; depending on the response criteria, about 40-60% of these patients recover (Prudic et al., 1996; van den Broek et al., 2004). However, this implies that approximately 50% of these patients do not respond or respond insufficiently. Furthermore, of the patients who initially recover on ECT, 40-80% relapse within half a year, which is in part dependent on the type of continuation treatment given after ECT (Sackeim et al., 2001).

A possible cause for less adequate response to ECT and the high relapse rate is that it does not alter the underlying negative cognitive schemas present in MDD. These schemas are relatively stable representations of previous knowledge and experiences that give rise to negative interpretations of current experiences and focus thoughts on negative aspects about oneself. Cognitive theories of depression suggest that these schemas thereby contribute to the development and maintenance of depression (Beck and Clark, 1988). When these negative schemas remain intact after treatment with ECT, it is not surprising that ECT alone is not enough.

Reconsolidation

Classic memory theories assumed that once memories have been stored, or consolidated, they cannot be erased and are resistant to change. New insights from reconsolidation theory posit that when memories are reactivated they become labile and modifiable and can be reconsolidated in another form. It is thus possible to weaken or even erase earlier memories when the re-consolidation process is complicated (Nader et al., 2000). This theory has a large influence on recent memory research, but was not new. Misanin and colleagues (1968) reported already in the late 1960’s that consolidated memories can be distorted in rodents when memories are reactivated prior to administration of an electroconvulsive shock (ECS), the animal variant of ECT. However, reconsolidation theory only became widely accepted after Nader and colleagues in 2000 showed that memories are disrupted when a new protein synthesis-dependent consolidation process is disrupted.

The first indication that memories in humans may be disrupted comes from research with ECT. Based on the animal experiments of Misanin and colleagues, Rubin (1976) reasoned: “If the patient’s attention is strongly directed, by hypnosis if necessary, to his most disturbing feelings and imagery, and if he is instantly given ECT (awake) there should result a

significantly greater amelioration and reduction of symptoms than is obtained when ECT is given in the usual way” (p. 88). To test this, he treated 28 patients with various psychiatric disorders. In patients with obsessions and delusions, symptoms were evoked during hypnosis prior to administration of ECT. In patients with compulsions and hallucinations, ECT was applied when symptoms occurred. In all these patients, of which 7 had received unsuccessful regular treatment with ECT previously, even one such ECT session led to "dramatical" improvements that persisted three months to at least 10 years.

The study of Rubin (1976) was very promising, but also not without problems. Rubin himself concluded that better controlled experiments were needed to confirm the results. But the main problem was that ECT was performed without anesthesia. It is also unclear whether, and under what circumstances, informed consent of the patients was obtained.
In addition, another study reported in the same year questioned whether reactivation of memories in humans could alter memories. Squire and colleagues (1976) asked participants to memorize pictures and combinations that were or were not reactivated prior to ECT, and the results suggested that the reactivation procedure did not influence memory. It is unclear why the work of Rubin was not pursued thereafter, but it was only until 2003 that the study was first referred to after its publication.

Recently, Kindt and colleagues (2009) showed that memory can be disrupted by reactivating memories after the administration of the beta-adrenergic receptor blocker propranolol. Importantly, we found that memory is worse when a story is reactivated prior to ECT than when it is not reactivated (Kroes et al., 2013). This demonstrates that memory reactivation prior to ECT can indeed weaken specific memories.

Schema-ECT

The combination of memory reactivation prior to ECT provides new opportunities for the treatment of severe depression. Specifically, it might be an effective method to weaken negative schemas that are not altered by ECT alone. According to the cognitive theory of depression this would ensure that current experiences are no longer automatically interpreted from a negative perspective, which would thereby contribute to a more positive self-image (Beck & Clark, 1988). This theory also predicts that weakening of negative schemas will lead to the recovery from depression and will reduce the risk to relapse. This may thereby provide a novel treatment option for patients that do not respond sufficiently to antidepressant or psychotherapy and then receive ECT, which might enhance efficacy of ECT at the short-term and might also provide patients better protection against relapse with a perspective of long-term recovery instead of persistent vulnerability (Bockting et al., 2005).

The purpose of this research is to study the effectiveness of schema-ECT in patients with severe depression. Patients with MDD that are indicated for ECT are randomized to schema-ECT or control-ECT. ECT itself is conducted as usual according to national guidelines (Nederlandse Vereniging voor Psychiatrie, 2010). In the schema group, ECT is preceded by a structured interview using the arrow-down technique that is part of cognitive-behavioral therapy (CBT) to reactivate negative schemas. In the control condition, patients will be interviewed about details that are also of clinical relevance but are not expected to activate

depressive schemas (e.g., their medical record, diet, exercise). Treatment efficacy is measured with the Hamilton Rating Scale for Depression (HAM-D) at baseline, every 6 sessions thereafter and after discontinuing ECT or after lowering the biweekly ECT session frequency. Changes in negative schemas are measured with the Dysfunctional Attitude Scale (DAS) and the Automatic Thoughts Questionnaire (ATQ) at the same timepoints. Furthermore, we will use the Self-Referent Encoding Task (SRET) before and after treatment to evaluate whether schema-ECT influences the processing of and memory for negative self-descriptions, as the SRET is thought to measure the influence of underlying negative schemas on cognition (Segal, 1988).

Neuroimaging biomarkers

In addition to determining the efficacy of schema-ECT, we aim to investigate how treatment response affects neuroimaging biomarkers and use these biomarkers to predict the response to ECT. We recently found in a small group of patients that MRI scans can predict the response to ECT with 85% sensitivity and specificity (van Waarde et al., submitted). To replicate and extend our findings to a larger and independent cohort and further refine our prediction accuracy using more neuroimaging parameters, we will obtain selected neuroimaging biomarkers at baseline. In addition, we will investigate the neural basis underlying the response to ECT using MRI. A leading hypothesis is that the antidepressant effects of various treatments are mediated by neurogenesis (Duman et al., 1997; Duman and Monteggia, 2006; Martinowich et al., 2007). Recently, it has become feasible to detect a marker of neurogenesis in vivo using magnetic resonance spectroscopy (MRS) (Manganas et al., 2007), and animal studies have shown that electroconvulsive stimulation (ECS) is the most potent stimulator of neurogenesis (Malberg et al., 2000). In line with this hypothesis, a preliminary study in a small group of patients reported that ECT increases hippocampal volume but the study did not control for nonspecific time effects, nor for stability of these effects after prolonged follow-up or when patients have a relapse (Nordanskog et al., 2010).

To test the hypothesis that the efficacy of ECT is mediated by neurogenesis, we will repeatedly measure hippocampal volumes and use magnetic resonance spectroscopy (MRS). We therefore aim to scan patients before and after the course of ECT and test for stability of changes or the re-occurrence of abnormalities when a relapse occurs. Therefore we will apply a third MRI-scan 3 months after completion of the acute-phase ECT course (or before the initiation of a new ECT-course when a relapse occurs). To further test whether this also alters the integration of the hippocampus within the frontolimbic circuitry that is thought to underlie depression at the neural systems level (Price and Drevets, 2010; MacQueen and Frodl, 2011), we will measure structural connectivity using diffusion tensor imaging (DTI) and resting-state functional connectivity using functional magnetic resonance imaging (fMRI). We hypothesize that the response to ECT is associated with increased neurogenesis and increased hippocampal connectivity with the prefrontal cortex in ECT responder compared to ECT non-responders.

In addition to the above mentioned neuroimaging biomarkers, MDD and especially severe and treatment resistant depression is seen as a disease with impairment of (behavioural)

reinforcement-learning and reward, characterized by dysfunctional fronto-limbic and/or cortico-striatal brain-circuits. For these processes, dopamine is essential (Fiorillo, 2008;Schultz, 2007). Most dopamine producing neurons are located in brainstem nuclei, of which the ventral tegmental area (VTA) is the most important for reinforcement-learning and reward. Projection pathways of the axons arising from the VTA amongst others consist of the mesolimbic pathway. It projects to the ventral striatum (including the nucleus accumbens), bed nucleus of the stria terminalis, hippocampus, amygdala, and septum. This circuitry is particularly important for motivation, the experience of pleasure, and reward (Dunlop, 2007), of which absence is an important predictor for insufficient response to antidepressive treatment. In one of the participating centers (UMCG Groningen), we have an advanced fMRI setup that investigates the VTA-ventral striatum circuitry by using primary rewards (apple-juice) in a probalistic learning task. Therefore, at the UMCG we will investigate whether alterations in baseline VTA-ventral striatum circuitry of the mesolimbic dopaminergic pathway can be used as a neuroimaging biomarker to predict the response to ECT and in addition, analyse the effect of ECT on this dopaminergic circuitry in the remaining 20 patients to be included in the UMCG.

Blood biomarkers

A related aim of this project is to find suitable blood biomarkers indicative of treatment response vs. non-response in ECT treatment. In line with the neurogenesis hypothesis, application of ECS in animals has shown that BDNF levels increase within the hippocampus after ECS treatment (Minelli et al., 2011).Others showed that ECT has also possible beneficial effects on dopaminergic neurons in a rat model for Parkinson's disease where the application of ECS prevented dopaminergic neuron loss by rescuing the expression of glial cell-line derived neurotropic factor (GDNF) (Anastasia et al., 2007), and evidence sugggests that ECT is effective in Parkinson’s patients (Pintor et al., 2012). ECT studies in both human and animals have shown that ECT also has effects on vaso endothelin growth factor (VEGF). Within animal studies ECS is associated with increasing levels of VEGF activating the mTOR pathway (Elfving and Wegener, 2012). In humans, it was recently shown that ECT treatment resulted in an increase of VEGF levels one month after the last treatment compared against the start of ECT treatment (Minelli et al., 2011). Both results may be associated with another study which showed that ECS results in an increased vascular density within the hippocampus as result of increased endothelial cell proliferation (Hellsten et al., 2004). Neurodegeneration is most often accompanied by massive glial activation were the activation of microglia is the most important one. Microglia are specialized immune cells protecting the brain against all kinds of damage. It was shown that ECT also has effects on microglia by activating them, possibly as an adaptation mechanism to a new equilibrium for neurotransmitters (Jansson et al., 2009). To investigate whether these and other biomarkers that are associated with depression (see Appendix I) are predictive of treatment response and how the levels of these biomarkers change with the response to ECT, we will collect blood samples from the venous catheter that is placed as part of the ECT procedure during the treatment phase or venapuncture for the follow-up samples.

2. OBJECTIVES

Primary Objectives:

To determine whether schema-ECT, in contrast to control-ECT

- Increases the remission rate of ECT
- Reduces the relapse rate during 24 weeks after the last ECT session
- Weakens negative schemas

Secondary Objectives

To find neurobiological markers that predict and are associated with the (sustained) response to ECT using:

- Hippocampal magnetic resonance spectroscopy (MRS)
- Structural connectivity using diffusion tensor imaging (DTI)
- Functional connectivity using functional magnetic resonance imaging (fMRI)
- Dopaminergic (dys-)function measured by a functional MRI of a probabilistic reward-related learning task (UMCG only)
- Blood samples

3. STUDY DESIGN

Our primary objective will be studied using a randomized controlled trial (RCT) comparing schema-ECT with control-ECT. ECT is performed according to the national guidelines, which consists in most cases of a minimum of 6 biweekly sessions until remission or a plateau in response is achieved for two weeks.

For our secondary objective to find neuroimaging and blood biomarkers that predict and are associated with ECT response, we will use a longitudinal parallel group design comparing later ECT responders with non-responders.

4. STUDY POPULATION

4.1 Population (base)

The study population exists of patients with an episode of MDD according to the DSM-IV and a clinical indication for ECT.

Considering the inclusion and exclusion criteria below, we expect 15 eligible patients per year at the AMC, 15 at the UMCG, and 25 at Rijnstate Hospital. Based on these expectations and a total sample of 98 (see below), the AMC will recruit 27 patients, the UMCG 27, and the Rijnstate Hospital 44. However, the actual recruitment and relative contribution of these centers may vary dependent on the actual recruitment rates.

4.2 Inclusion criteria

- Diagnosis of major depressive disorder (MDD) without psychotic symptoms assessed with the Mini Neuropsychiatric Interview (MINI)
- Clinical indication for ECT
- 18-70 years of age
- Willingness and ability to give written informed consent and willingness and ability to understand, to participate and to comply with the study requirements

4.3 Exclusion criteria

- Bipolar disorder, schizophrenia, primary alcohol or drug abuse, or any cognitive disorder as assessed with the Mini Neuropsychiatric Interview (MINI)
- Patients with MR contraindications such as metal implants or claustrophobia will be excluded from the neuroimaging study

4.4 Sample size calculation

Results from a recent study in a clinically representative ECT population in The Netherlands showed that 42% of medication resistant depressed patients achieve remission (van Waarde et al., 2012). According to the recommendations of Cohen (Cohen, 1988), the corresponding total sample size to detect a higher remission rate with medium effect size (25%), 80% power and a one-tailed alpha of 5% is 98 (Cohen, 1992).

5. TREATMENT OF SUBJECTS

5.1 Investigational product/treatment

The subjects will be randomised to schema-ECT or control-ECT. ECT is conducted as usual according to national guidelines in both patient groups (Nederlandse Vereniging voor Psychiatrie, 2010). In the schema group, ECT is preceded by a structured interview using the arrow-down technique, a technigue known from cognitive-behavioral therapy (CBT) to reactivate negative schemas (see appendix II). In the control condition, patients will be interviewed about details that are also of clinical relevance but are not expected to activate depressive schemas (i.e., their medical record, diet, exercise).

5.2 Use of co-intervention (if applicable)

Apart from ECT, patients are often treated with antidepressants and/or second generation antipsychotic drugs (used as augmentation strategy; (Nelson and Papakostas, 2009)). This treatment will be kept as usual and no specific instructions will be given to taper these drugs because of the study. Benzodiazepines and anticonvulsants will be tapered as usual, because of unwanted seizure suppressive properties of these drugs.

5.3 Escape medication (if applicable)

N/A

6. INVESTIGATIONAL PRODUCT

N/A

6.1 Name and description of investigational product(s)

6.2 Summary of findings from non-clinical studies

6.3 Summary of findings from clinical studies

6.4 Summary of known and potential risks and benefits

6.5 Description and justification of route of administration and dosage

6.6 Dosages, dosage modifications and method of administration

6.7 Preparation and labelling of Investigational Medicinal Product

6.8 Drug accountability

7. NON-INVESTIGATIONAL PRODUCT

N/A

7.1 Name and description of non-investigational product(s)

7.2 Summary of findings from non-clinical studies

7.3 Summary of findings from clinical studies

7.4 Summary of known and potential risks and benefits

7.5 Description and justification of route of administration and dosage

7.6 Dosages, dosage modifications and method of administration

7.7 Preparation and labelling of Non Investigational Medicinal Product

7.8 Drug accountability

8. METHODS

8.1 Study parameters/endpoints

8.1.1 Main study parameter/endpoint

- Remission rate defined as Hamilton Rating Scale for Depression (HAM-D) score ≤ 7 (Frank et al., 1991)
- Treatment response defined as at least 50% reduction in HAM-D score (Frank et al., 1991)
- Change in Dysfunctional Attitude Scale (DAS) score
- Change in Automatic Thoughts Questionnaire (ATQ) score
- Change in Self-Referent Encoding Task (SRET) performance
- Time to relapse/recurrence
  Relapse/recurrence will be defined as
  - HAM-D score ≥ 15 after obtaining remission (HDRS≤7) maintained for at least two weeks OR
  - increase in HAM-D score with ≥ 10 points after an initial response (minimal ≥50% HAM-D decrease) maintained for at least two weeks OR
  - leading to a restart of ECT (twice weekly)

8.1.2 Secondary study parameters/endpoints (if applicable)

- Hippocampal magnetic resonance spectroscopy (MRS)
- Structural connectivity using diffusion tensor imaging (DTI)
- Functional connectivity using functional magnetic resonance imaging (fMRI)
- Functional MRI of a probabilistic reward-related learning task
- Biomarker levels determined from blood samples

8.1.3 Other study parameters (if applicable)

- Premorbid IQ as measured with the Dutch Adult Reading Test (Nederlandse Leestest voor Volwassenen)
- General cognitive function as measured with the Mini Mental State Examination (MMSE)
- Global Assessment of Functioning (GAF)
- Clinical Global Impression (CGI)
- Number of successful ECT sessions (motor-seizure>20s) until remission
- Electrode placement at end of ECT
- Concomitant medication (for analyses we will standardize dosages according to dose equivalents of benzodiazepines (diazepam), antidepressants (nortriptyline) and antipsychotics (haloperidol))

8.2 Randomisation, blinding and treatment allocation

Subjects will be randomized to schema-ECT or control-ECT, stratified for research site.

8.3 Study procedures

Study design

Patients that receive a clinical indication for ECT are requested to participate in the study. Subjects that fulfil the inclusion and exclusion criteria and sign informed consent are randomized to schema-ECT or control-ECT. ECT itself is performed as usual according to the national guidelines. In the schema-ECT group, the ECT protocol is preceded by a structured interview using the arrow-down technique that is part of cognitive-behavioral therapy (CBT) to reactivate negative schemas (see appendix II). In the control-ECT, patients will be interviewed about details that are also of clinical relevance but are not expected to activate depressive schemas (e.g., their medical record, diet, exercise).

Pre-treatment baseline measures are obtained before start of ECT. These contain HAM-D, DAS and ATQ scores, the SRET, and the neuroimaging session. The level of treatment resistance will be quantified with a modification of the Maudsley Staging Method for treatment resistance (Fekadu et al., 2009; Ruhe et al., 2012). During treatment, HAM-D scores are obtained at least every 6 sessions to follow treatment response. Within one week after the last ECT session (and before the transition of possible maintenance ECT (<1 session per week)), all parameters including the MRI-assessment are assessed again as post-treatment measurement. Thereafter, patients are evaluated at 2-week intervals the first 8 weeks, and at 4-week intervals for the remaining 12 weeks to determine possible treatment relapse. In order to investigate long-term stability of changes we will repeat the MRI-assessment at 3 months after the termination of the acute-phase ECT-series (i.e. after the last ECT session but irrespective of the transition to possible maintenance ECT). In case a patient has a relapse before these three months, we will ask for a third MRI-scan at 3 months likewise, unless the patient starts a new series of ECT, then the MRI will be planned before the start of the new ECT-serie.s.

Schema reactivation

Negative schemas will be reactivated approximately 30 min. prior to the application of ECT. Schema-ECT consists of reactivation of depressive schemas using the arrow-down technique that is part of cognitive-behavioral therapy (CBT). After inclusion of the study, negative schemas will be measured using the Dysfunctional Attitude Scale (DAS; see below). Together with the patient, an experienced therapist will discuss the depressive cognitions that are indicated by the patient to retrieve occasions that led to these depressive cognitions. Six different events will be retrieved in detail for which a protocol will be drafted that will form the basis for different negative schema reactivations in different ECT sessions. In the control condition, patients will be

interviewed about details that are also of clinical relevance but are not expected to activate depressive schemas (e.g., their medical record, diet, exercise).

ECT

ECT will be performed according to the national ECT guidelines (Nederlandse Vereniging voor Psychiatrie, 2010) and a recent study (van Waarde et al., 2012). ECT will be administered using a constant-current (0.9 Ampère), brief-pulse (0.5 ms in right unilateral (RUL) ECT; 0.5 ms in bifrontotemporal (BL) ECT) device (Thymatron IV; Somatics Incorporation, Lake Bluff, Illinois, USA), after intravenous induction of anesthesia with etomidate (0.2 mg/kg body mass), muscle paralysis with succinylcholine (0.5–1 mg/kg body mass), and appropriate oxygenation (100 % oxygen, positive pressure) until the resumption of spontaneous respiration.
ECT will start with 6 sessions RUL electrode placement, except if the clinician decides to start with BL placement due to the clinical condition of the patient or previous effective BL treatment. After insufficient response (<50% improvement) after 6 ECT-sessions, RUL electrode placement is switched to BL placement. During the first session, the seizure threshold (ST) will be measured by an internationally accepted, empirical, age-adjusted, titration method. If the starting stimulus dose failed to elicit a seizure of at least 15s of motor activity measured with the cuff method or ≥25 s on EEG, stimulus charge will be increased according to the titration schedule (for patients aged >50 years: 25.2, 50.4, 100.8, 201.6, and 403.2 mC; for patients aged <50 years: 50.4, 100.8, 201.6, and 403.2 mC), and the patient will be restimulated after 30 s. After the titration session, the dosage is set at 6 times the ST for RUL treatment and at 2.5 times the ST for BL.
ECT is discontinued when remission is achieved, or when no further improvement is observed over a period of 2 weeks. ECT is considered to be discontinued when patients show no improvement after 10 BL sessions, but the clinician may decide to continue ECT which implies continuation of the study.

Negative schemas

The influence schema-ECT on negative schemas and automatic thoughts will be measured with the Dysfunctional Attitude Scale (DAS; (Weissman and Beck, 1978) and the Automatic Thoughts Questionnaire (ATQ; (Hollon and Kendall, 1980). To assess whether schema-ECT also influences the processing of and memory for depression-relevant adjectives the Self-Referent Encoding Task (SRET) will be used, which will be applied before and after the full ECT-course. The SRET measures the influence of underlying negative schemas on cognitions (Segal, 1988). Sixteen depressive and 16 non-depressive adjectives will be presented for 3s. Participants are requested to indicate whether the adjective describes themselves or not. After the initial test, participants are requested to recall as many words as possible. The dependent measures are reaction times, the number of words endorsed as self-descriptive, and the number of recalled words (Dobson and Shaw, 1987).

Neuroimaging

MR scanning will be performed on a 3 Tesla Philips MR scanner. The following scans will be acquired:

- Structural scans using 3D-MPRAGE with 1.0 x 1.0 x 1.0 mm^3 voxels
- Resting-state fMRI scans using GE-EPI with 3.0 x 3.0 x 3.0 mm^3 voxels with TR = 2.0 s and TE = 27 ms
- DTI scans with 2.0 x 2.0 x 2.0 mm^3 voxels, 46 directions and b = 1000
- Single-voxel ^{1}H-MRS aligned along the hippocampal axis with 30 x 12 x 12 mm^3 voxel, TE = 30 ms, TR = 2.0 s and 128 averages.
- (UMCG only) Reward-related learning fMRI: After staying thirsty overnight, and refraining from smoking for the same period, a Pavlovian-learning paradigm will be used, delivering 3 blocks of 15 trials in which small amounts (0.2ml) of liquid (rewarding apple -juice) at different probabilities (80%-20%) after 2 conditional stimuli. One block (30 trials) without juice delivery but with the conditional stimuli (unconditioned yet) will precede these blocks. With the changing probabilities of water delivery temporal difference reward-learning signals will be calculated which will be used as a regressor of interest in the analyses of interest in the analyses of BOLD-responses.

Blood sampling

At the following ECT-sessions: 1, 3, 6 (and possibly session 9 and 12 in case more ECT sessions are needed), approximately 45 ml of blood per session will be withdrawn. Two weeks and 3 months after the last ECT treatment, last blood samples will be taken. Sampling takes place from the venous catheter which is already present for anaesthesia induction in patients. At the indicated ECT-sessions small samples of blood will be withdrawn immediately before ECT induction, at 0.5 and 1 hrs afterwards (15 ml each timepoint; 5 ml serum clotting tubes, 5 ml EDTA tubes, 5 ml heparine tubes), and from the first patient at every ECT session an extra blood sample will be taken 2 hrs after ECT induction (these patients will remain at the post-operative care unit long enough after their ECT, so a 2 hour measurement is feasible). For the purpose of this study we might have to use slightly thicker needles in the i.v. cannulas, as otherwise blood-collection might not be possible. When withdrawal from the cannula is not possible, a vena-puncture will be done if the patient agrees. Blood samples taken 2 weeks and 3 months after the last treatment, will be taken by venapuncture. Samples will be collected in a serum separation tube, a heparine tube and an EDTA tube (5 ml each). Samples will be stored at -80 degrees Celsius until analysed.

8.4 Withdrawal of individual subjects

Subjects can leave the study at any time for any reason if they wish to do so without any consequences. The investigator can decide to withdraw a subject from the study for urgent medical reasons or for the regular clinical criteria to terminate an ECT

course such as an insufficient response after sufficient treatment or severe cognitive side-effects that outweigh the clinical benefit.

8.4.1 Specific criteria for withdrawal (if applicable)

N/A

8.5 Replacement of individual subjects after withdrawal

N/A

8.6 Follow-up of subjects withdrawn from treatment

Patients withdrawn from ECT will be followed by their treating physician according to usual clinical practice. If the patient agrees on additional measures, we will continue the protocol for relapse identification unaltered.

8.7 Premature termination of the study

N/A

9. SAFETY REPORTING

9.1 Temporary halt for reasons of subject safety

In accordance to section 10, subsection 4, of the WMO, the sponsor will suspend the study if there is sufficient ground that continuation of the study will jeopardise subject health or safety. The sponsor will notify the accredited METC without undue delay of a temporary halt including the reason for such an action. The study will be suspended pending a further positive decision by the accredited METC. The investigator will take care that all subjects are kept informed.

9.2 AEs, SAEs and SUSARs

9.2.1 Adverse events (AEs)

Adverse events are defined as any undesirable experience occurring to a subject during the study, whether or not considered related to the experimental intervention. All adverse events reported spontaneously by the subject or observed by the investigator or his staff will be recorded.

9.2.2 Serious adverse events (SAEs)

A serious adverse event is any untoward medical occurrence or effect that at any dose:

- results in death;
- is life threatening (at the time of the event);
- requires hospitalisation or prolongation of existing inpatients' hospitalisation;
- results in persistent or significant disability or incapacity;
- is a congenital anomaly or birth defect;
- Any other important medical event that may not result in death, be life threatening, or require hospitalization, may be considered a serious adverse experience when, based upon appropriate medical judgement, the event may jeopardize the subject or may require an intervention to prevent one of the outcomes listed above.

In case of an SAE the patient will be withdrawn from the study, and the treating physician will initiate further action according to the type of SAE.

The sponsor will report the SAEs through the web portal *ToetsingOnline* to the accredited METC that approved the protocol, within 15 days after the sponsor has first knowledge of the serious adverse reactions.

SAEs that result in death or are life threatening should be reported expedited. The expedited reporting will occur not later than 7 days after the responsible investigator has first knowledge of the adverse reaction. This is for a preliminary report with another 8 days for completion of the report.

9.2.3 Suspected unexpected serious adverse reactions (SUSARs)

N/A

9.3 Annual safety report

The sponsor will submit, once a year throughout the clinical trial, a safety report combined with the annual progress report (see 12.4) to the accredited METC.

This safety report consists of:

- a list of all serious adverse events;
- a report concerning the safety of the subjects consisting of an evaluation of the balance between the efficacy and the harmfulness of the treatment under investigation.

9.4 Follow-up of adverse events

All AEs will be followed until they have abated, or until a stable situation has been reached. Depending on the event, follow up may require additional tests or medical procedures as indicated, and/or referral to the general physician or a medical specialist. SAEs need to be reported till end of study within the Netherlands, as defined in the protocol.

9.5 Data Safety Monitoring Board (DSMB) / Safety Committee

N/A

10. STATISTICAL ANALYSIS

10.1 Primary study parameter(s)

Efficacy data will be analyzed in an intention-to-treat and per-protocol analysis. Remission rates will be presented as proportions and following the recommendations of Cohen (Cohen, 1988) will be tested using the normal curve test with arcsine transformation. Time to relapse will be presented and analyzed using Kaplan-Meier survival analysis and log-rank test. Other parameters are expected to be normally distributed and will be analyzed using ANOVA. Alpha will be set at 5%.

10.2 Secondary study parameter(s)

Resting-state fMRI data will be analyzed using seed-based correlation analysis in SPM and voxel-wise statistical tests will be corrected for multiple comparisons. DTI tractography of the uncinate fasciculus will be performed in DTI studio. MRS data will be analyzed using singular value decomposition as described in (Manganas et al., 2007). Tractography and MRS data will be analyzed using ANOVA. For the reinforcement learning paradigm, a temporal difference error-signal approach will be used as described by Kumar et. al, (2008) to analyse the dopaminergic BOLD response in VTA/ventral striatum/habenula ROIs. Alpha will be set at 5%.

The potential biomarkers listed in Appendix I will first be compared in linear mixed models for individual biomarkers in association with clinical changes. Furthermore, we will also apply data-driven approaches (like support vector machine-learning) to recognize profiles of these proteins (and their changes directly after the ECT and over time of subsequent sessions) in association with changes in clinical status. Alpha will be set at 5%.

10.3 Other study parameters

Other parameters will be analyzed using ANOVA or nonparametric tests where appropriate. Alpha will be set at 5%.

10.4 Interim analysis (if applicable)

To avoid unnecessary exposure of patients to unexpected averse effects of the treatment once there is sufficient evidence, we will perform an interim analysis. It is possible to detect a large effect with 80% power at trend level (alpha = 10%) with 19 subjects per group (Cohen, 1992). Therefore, we will perform an interim analysis when 38 patients have been included.

11. ETHICAL CONSIDERATIONS

11.1 Regulation statement

This study will be conducted according to the principles of the Declaration of Helsinki and in accordance with the Medical Research Involving Human Subjects Act (WMO).

11.2 Recruitment and consent

Patients referred for ECT will be asked by their treating physician to participate after explaining the study. Interested patients will receive the information brochure and have at least a day to consider their participation. Informed consent will be obtained before participation by the investigator.

Healthy controls will be recruited via advertisements. They will receive the information brochure and have at least a day to consider their participation. They will receive further information by the investigator who will also ask them to sign informed consent.

11.3 Objection by minors or incapacitated subjects (if applicable)

N/A

11.4 Benefits and risks assessment, group relatedness

The burden of ECT is considerable but is warranted for otherwise treatment resistant patients that suffer from severe depression. Shortly after treatment, minor side effects can occur such as headache, nausea, muscle ache, forgetfulness and confusion. The risks associated with ECT are of cardiovascular nature due to increased cerebral blood flow, which is why ECT is not preferred for patients with pheochromocytoma, recent myocardial infarction (<4 weeks), instable angina pectoris (NYHA class IV), recent cerebrovascular accident or intracranial surgery (<3 months). For eligible patients, the risk of ECT can be considered negligible. However, there are no absolute contraindications and the risk associated with depression (e.g., suicidality) may outweigh the cardiovascular risks and such patients may nevertheless be included. Importantly, only the regular ECT-population will be recruited for this study.

The additional burden for participating in this study is minimal and the additional risk can be considered negligible. Besides the potential benefit for the population of treatment resistant depressive patients, the patients may also benefit from participating themselves as we expect that remission and relapse rates will be beneficial in the schema-ECT group. The additional burden for participating in the neuroimaging study can be

considered minimal, and the additional risk for participants without MRI contraindications is negligible.

11.5 Compensation for injury

The sponsor/investigator has a liability insurance which is in accordance with article 7, subsection 9 of the WMO.

The sponsor (also) has an insurance which is in accordance with the legal requirements in the Netherlands (Article 7 WMO and the Measure regarding Compulsory Insurance for Clinical Research in Humans of 23th June 2003). This insurance provides cover for damage to research subjects through injury or death caused by the study.

1. € 450.000,-- (i.e. four hundred and fifty thousand Euro) for death or injury for each subject who participates in the Research;
2. € 3.500.000,-- (i.e. three million five hundred thousand Euro) for death or injury for all subjects who participate in the Research;
3. € 5.000.000,-- (i.e. five million Euro) for the total damage incurred by the organisation for all damage disclosed by scientific research for the Sponsor as ‘verrichter’ in the meaning of said Act in each year of insurance coverage.

The insurance applies to the damage that becomes apparent during the study or within 4 years after the end of the study.

11.6 Incentives (if applicable)

Patients will receive 20 Euros compensation for the randomized clinical trial (RCT) and 20 Euros compensation for the MRI study. The inclusion and consent for the MRI study is separated from the RCT because not every patient that is eligible for the RCT will also be eligible for the MRI study, and the sample size for the MRI study can be smaller than for the RCT. Healthy volunteers will receive 40 Euros for their participation. The compensation is on the basis of full participation upon completion of the study. In the case of premature withdrawal from the study for other than safety reasons, participants will be partially reimbursed according to their time investment.

12. ADMINISTRATIVE ASPECTS, MONITORING AND PUBLICATION

12.1 Handling and storage of data and documents

Data will be coded according to the site of investigation and enrolment (ECT-AMC/ARN/GRO-01) and handled confidentially. The data will be stored for 20 years and the code will be safeguarded by the investigator. Participants will be asked if we may use the data for additional research with a similar research question.

12.2 Monitoring and Quality Assurance

N/A

12.3 Amendments

Amendments are changes made to the research after a favourable opinion by the accredited METC has been given. All amendments will be notified to the METC that gave a favourable opinion.

12.4 Annual progress report

The sponsor/investigator will submit a summary of the progress of the trial to the accredited METC once a year. Information will be provided on the date of inclusion of the first subject, numbers of subjects included and numbers of subjects that have completed the trial, serious adverse events/ serious adverse reactions, other problems, and amendments.

12.5 End of study report

The investigator will notify the accredited METC of the end of the study within a period of 8 weeks. The end of the study is defined as the last patient’s last visit.

In case the study is ended prematurely, the investigator will notify the accredited METC within 15 days, including the reasons for the premature termination.

Within one year after the end of the study, the investigator/sponsor will submit a final study report with the results of the study, including any publications/abstracts of the study, to the accredited METC.

12.6 Public disclosure and publication policy

The trial will be registered at www.nederlandstrialregister.nl before recruitment of the first patient, and the results of the study will be disclosed without reservation.

13. STRUCTURED RISK ANALYSIS

N/A

13.1 Potential issues of concern

13.2 Synthesis

14. REFERENCES

Alder J, Thakker-Varia S, Bangasser DA, Kuroiwa M, Plummer MR, Shors TJ, Black IB (2003) Brain-derived neurotrophic factor-induced gene expression reveals novel actions of VGF in hippocampal synaptic plasticity. J Neurosci 23:10800-10808.
Anastasia A, de Erausquin GA, Wojnacki J, Masco DH (2007) Protection of dopaminergic neurons by electroconvulsive shock in an animal model of Parkinson's disease. J Neurochem 103:1542-1552.
Asheuer M, Pflumio F, Benhamida S, Dubart-Kupperschmitt A, Fouquet F, Imai Y, Aubourg P, Cartier N (2004) Human CD34+ cells differentiate into microglia and express recombinant therapeutic protein. Proc Natl Acad Sci U S A 101:3557-3562.
Beck AT, Clark DA (1988) Anxiety and depression: an information processing perspective. Anxiety Stress Coping 1:23-36.
Bockting CL, Schene AH, Spinhoven P, Koeter MW, Wouters LF, Huyser J, Kamphuis JH (2005) Preventing relapse/recurrence in recurrent depression with cognitive therapy: a randomized controlled trial. J Consult Clin Psychol 73:647-657.
Burg MM, Martens EJ, Collins D, Soufer R (2011) Depression predicts elevated endothelin-1 in patients with coronary artery disease. Psychosom Med 73:2-6.
Choi J, Lee HW, Suk K (2011) Increased plasma levels of lipocalin 2 in mild cognitive impairment. J Neurol Sci 305:28-33.
Cohen J (1988) Statistical power analysis for the behavioral sciences, 2nd ed. Edition. Hillsdale: Lawrence Erlbaum Associates.
Cohen J (1992) A power primer. Psychol Bull 112:155-159.
Dobson KS, Shaw BF (1987) Specificity and stability of self-referent encoding in clinical depression. J Abnorm Psychol 96:34-40.
Duman RS, Monteggia LM (2006) A neurotrophic model for stress-related mood disorders. Biol Psychiatry 59:1116-1127.
Duman RS, Heninger GR, Nestler EJ (1997) A molecular and cellular theory of depression. Arch Gen Psychiatry 54:597-606.
Dunlop BW, Nemeroff CB (2007a): The role of dopamine in the pathophysiology of depression. Arch Gen Psychiatry 64: 327-337.
Elfving B, Wegener G (2012) Electroconvulsive seizures stimulate the vegf pathway via mTORC1. Synapse 66:340-345.
Fekadu A, Wooderson SC, Markopoulou K, Cleare AJ (2009) The Maudsley Staging Method for treatment-resistant depression: prediction of longer-term outcome and persistence of symptoms. J Clin Psychiatry 70:952-957.
Feng P, Huang C (2013) Phospholipase D-mTOR signaling is compromised in a rat model of depression. J Psychiatr Res 47:579-585.
Fiorillo CD, Newsome WT, Schultz W (2008): The temporal precision of reward prediction in dopamine neurons. Nat Neurosci 11:966-73.
Frank E, Prien RF, Jarrett RB, Keller MB, Kupfer DJ, Lavori PW, Rush AJ, Weissman MM (1991) Conceptualization and rationale for consensus definitions of terms in major depressive disorder: remission, recovery, relapse, and recurrence. Arch Gen Psychiatry 48:851-855.
Galecki P, Galecka E, Maes M, Chamielec M, Orzechowska A, Bobinska K, Lewinski A, Szemraj J (2012) The expression of genes encoding for COX-2, MPO, iNOS, and sPLA2-IIA in patients with recurrent depressive disorder. J Affect Disord 138:360-366.
Gregg C, Shikar V, Larsen P, Mak G, Chojnacki A, Yong VW, Weiss S (2007) White matter plasticity and enhanced remyelination in the maternal CNS. J Neurosci 27:1812-1823.
Gutierrez H, Davies AM (2011) Regulation of neural process growth, elaboration and structural plasticity by NF-kappaB. Trends Neurosci 34:316-325.
Heijnen WT, Birkenhager TK, Wierdsma AI, van den Broek WW (2010) Antidepressant pharmacotherapy failure and response to subsequent electroconvulsive therapy: a meta-analysis. J Clin Psychopharmacol 30:616-619.

Heller RA, Kronke M (1994) Tumor necrosis factor receptor-mediated signaling pathways. J Cell Biol 126:5-9.
Hellsten J, Wennstrom M, Bengzon J, Mohapel P, Tingstrom A (2004) Electroconvulsive seizures induce endothelial cell proliferation in adult rat hippocampus. Biol Psychiatry 55:420-427.
Hestad KA, Tonseth S, Stoen CD, Ueland T, Aukrust P (2003) Raised plasma levels of tumor necrosis factor alpha in patients with depression: normalization during electroconvulsive therapy. J ECT 19:183-188.
Hollon SD, Kendall PC (1980) Cognitive self-statements in depression: development of an automatic thoughts questionnaire. Cognit Ther Res 4:383-395.
Jansson L, Wennstrom M, Johanson A, Tingstrom A (2009) Glial cell activation in response to electroconvulsive seizures. Prog Neuropsychopharmacol Biol Psychiatry 33:1119-1128.
Kanapeckiene V, Kalibatas J, Redaitiene E, Ceremnych J (2007) The association between cytomegalovirus infection and aging process. Medicina (Kaunas) 43:419-424.
Kindt M, Soeter M, Vervliet B (2009) Beyond extinction: erasing human fear responses and preventing the return of fear. Nat Neurosci 12:256-258.
Klok MD, Jakobsdottir S, Drent ML (2007) The role of leptin and ghrelin in the regulation of food intake and body weight in humans: a review. Obes Rev 8:21-34.
Koenig MK, Butler IJ (2011) Tetrahydrobiopterin deficiency. In: Oxidative stress and free radical damage in neurology (Godoth N, Gobel HH, eds), pp 225-234. New York: Springer.
Krishnan V, Nestler EJ (2008) The molecular neurobiology of depression. Nature 455:894-902.
Kroes MCW, Tendolkar I, van Wingen GA, van Waarde JA, Strange BA, Fernandez G (2013) An electroconvulsive therapy procedure impairs reconsolidation of emotional episodic memories in humans. Nat Neurosci:in press.
Kumar P, Waiter G, Ahearn T, Milders M, Reid I, Steele JD. Abnormal temporal difference reward-learning signals in major depression. Brain. 2008; 131: 2084-2093.
Kwak SS, Suk J, Choi JH, Yang S, Kim JW, Sohn S, Chung JH, Hong YH, Lee DH, Ahn JK, Min H, Fu YM, Meadows GG, Joe CO (2011) Autophagy induction by tetrahydrobiopterin deficiency. Autophagy 7:1323-1334.
Lin NY, Beyer C, Giessl A, Kireva T, Scholtysek C, Uderhardt S, Munoz LE, Dees C, Distler A, Wirtz S, Kronke G, Spencer B, Distler O, Schett G, Distler JH (2013) Autophagy regulates TNFalpha-mediated joint destruction in experimental arthritis. Ann Rheum Dis 72:761-768.
Liu Y, Ho RC, Mak A (2012) Interleukin (IL)-6, tumour necrosis factor alpha (TNF-alpha) and soluble interleukin-2 receptors (sIL-2R) are elevated in patients with major depressive disorder: a meta-analysis and meta-regression. J Affect Disord 139:230-239.
MacQueen G, Frodl T (2011) The hippocampus in major depression: evidence for the convergence of the bench and bedside in psychiatric research? Mol Psychiatry 16:252-264.
Malberg JE, Eisch AJ, Nestler EJ, Duman RS (2000) Chronic antidepressant treatment increases neurogenesis in adult rat hippocampus. J Neurosci 20:9104-9110.
Manganas LN, Zhang X, Li Y, Hazel RD, Smith SD, Wagshul ME, Henn F, Benveniste H, Djuric PM, Enikolopov G, Maletic-Savatic M (2007) Magnetic resonance spectroscopy identifies neural progenitor cells in the live human brain. Science 318:980-985.
Martinowich K, Manji H, Lu B (2007) New insights into BDNF function in depression and anxiety. Nat Neurosci 10:1089-1093.
Milanesi E, Minelli A, Cattane N, Cattaneo A, Mora C, Barbon A, Mallei A, Popoli M, Florio V, Conca A, Bignotti S, Gennarelli M (2012) ErbB3 mRNA leukocyte levels as a biomarker for major depressive disorder. BMC Psychiatry 12:145.
Minelli A, Zanardini R, Abate M, Bortolomasi M, Gennarelli M, Bocchio-Chiavetto L (2011) Vascular Endothelial Growth Factor (VEGF) serum concentration during electroconvulsive therapy (ECT) in treatment resistant depressed patients. Prog Neuropsychopharmacol Biol Psychiatry 35:1322-1325.
Misanin JR, Miller RR, Lewis DJ (1968) Retrograde amnesia produced by electroconvulsive shock after reactivation of a consolidated memory trace. Science 160:554-555.

Morris AA, Ahmed Y, Stoyanova N, Hooper WC, De Staerke C, Gibbons G, Quyyumi A, Vaccarino V (2012) The association between depression and leptin is mediated by adiposity. Psychosom Med 74:483-488.
Muller N, Myint AM, Schwarz MJ (2011) Inflammatory biomarkers and depression. Neurotox Res 19:308-318.
Nader K, Schafe GE, Le Doux JE (2000) Fear memories require protein synthesis in the amygdala for reconsolidation after retrieval. Nature 406:722-726.
Naude PJ, Nyakas C, Eiden LE, Ait-Ali D, van der Heide R, Engelborghs S, Luiten PG, De Deyn PP, den Boer JA, Eisel UL (2012) Lipocalin 2: novel component of proinflammatory signaling in Alzheimer's disease. FASEB J 26:2811-2823.
Nederlandse Vereniging voor Psychiatrie (2010) Richtlijn elektroconvulsietherapie: tweede herziene versie. Utrecht: De Tijdstroom.
Nelson JC, Papakostas GI (2009) Atypical antipsychotic augmentation in major depressive disorder: a meta-analysis of placebo-controlled randomized trials. Am J Psychiatry 166:980-991.
Nordanskog P, Dahlstrand U, Larsson MR, Larsson EM, Knutsson L, Johanson A (2010) Increase in hippocampal volume after electroconvulsive therapy in patients with depression: a volumetric magnetic resonance imaging study. J ECT 26:62-67.
Pintor LP, Valldeoriola F, Fernandez-Egea E, Sanchez R, Rami L, Tolosa E, Muniz A, Marti MJ, Bernardo M (2012) Use of electroconvulsive therapy in Parkinson disease with residual axial symptoms partially unresponsive to L-dopa: a pilot study. J ECT 28:87-91.
Price JL, Drevets WC (2010) Neurocircuitry of mood disorders. Neuropsychopharmacology 35:192-216.
Prudic J, Haskett RF, Mulsant B, Malone KM, Pettinati HM, Stephens S, Greenberg R, Rifas SL, Sackeim HA (1996) Resistance to antidepressant medications and short-term clinical response to ECT. Am J Psychiatry 153:985-992.
Redrobe JP, Dumont Y, Quirion R (2002) Neuropeptide Y (NPY) and depression: from animal studies to the human condition. Life Sci 71:2921-2937.
Rubin RD (1976) Clinical use of retrograde amnesia produced by electroconvulsive shock. A conditioning hypothesis. Can Psychiatr Assoc J 21:87-90.
Ruhe HG, van Rooijen G, Spijker J, Peeters FP, Schene AH (2012) Staging methods for treatment resistant depression. A systematic review. J Affect Disord 137:35-45.
Rush AJ, Trivedi MH, Wisniewski SR, Nierenberg AA, Stewart JW, Warden D, Niederehe G, Thase ME, Lavori PW, Lebowitz BD, McGrath PJ, Rosenbaum JF, Sackeim HA, Kupfer DJ, Luther J, Fava M (2006) Acute and longer-term outcomes in depressed outpatients requiring one or several treatment steps: a STAR*D report. Am J Psychiatry 163:1905-1917.
Sackeim HA, Haskett RF, Mulsant BH, Thase ME, Mann JJ, Pettinati HM, Greenberg RM, Crowe RR, Cooper TB, Prudic J (2001) Continuation pharmacotherapy in the prevention of relapse following electroconvulsive therapy: a randomized controlled trial. JAMA 285:1299-1307.
Schmidt HD, Shelton RC, Duman RS (2011) Functional biomarkers of depression: diagnosis, treatment, and pathophysiology. Neuropsychopharmacology 36:2375-2394.
Schroeder M, Krebs MO, Bleich S, Frieling H (2010) Epigenetics and depression: current challenges and new therapeutic options. Curr Opin Psychiatry 23:588-592.
Schultz W (2007): Multiple dopamine functions at different time courses. Annu Rev Neurosci 30: 259-288.
Segal ZV (1988) Appraisal of the self-schema construct in cognitive models of depression. Psychol Bull 103:147-162.
Shingo T, Gregg C, Enwere E, Fujikawa H, Hassam R, Geary C, Cross JC, Weiss S (2003) Pregnancy-stimulated neurogenesis in the adult female forebrain mediated by prolactin. Science 299:117-120.
Shukla L, Narayanaswamy JC, Gopinath S, Math SB (2012) Electroconvulsive therapy for the treatment of organic catatonia due to viral encephalitis. J ECT 28:e27-28.
Sperner-Unterweger B, Kohl C, Fuchs D (2012) Immune changes and neurotransmitters: Possible interactions in depression? Prog Neuropsychopharmacol Biol Psychiatry.

Squire LR, Slater PC, Chace PM (1976) Reactivation of recent or remote memory before electroconvulsive therapy does not produce retrograde amnesia. Behav Biol 18:335-343.
Stelzhammer V, Guest PC, Rothermundt M, Sondermann C, Michael N, Schwarz E, Rahmoune H, Bahn S (2012) Electroconvulsive therapy exerts mainly acute molecular changes in serum of major depressive disorder patients. Eur Neuropsychopharmacol.
Strandberg TE, Pitkala KH, Linnavuori K, Tilvis RS (2004) Cognitive impairment and infectious burden in the elderly. Arch Gerontol Geriatr Suppl:419-423.
Stuebe AM, Grewen K, Pedersen CA, Propper C, Meltzer-Brody S (2012) Failed lactation and perinatal depression: common problems with shared neuroendocrine mechanisms? J Womens Health (Larchmt) 21:264-272.
Tamatam A, Khanum F, Bawa AS (2012) Genetic biomarkers of depression. Indian J Hum Genet 18:20-33.
Uddin M, Koenen KC, Aiello AE, Wildman DE, de los Santos R, Galea S (2011) Epigenetic and inflammatory marker profiles associated with depression in a community-based epidemiologic sample. Psychol Med 41:997-1007.
van den Broek WW, de Lely A, Mulder PG, Birkenhager TK, Bruijn JA (2004) Effect of antidepressant medication resistance on short-term response to electroconvulsive therapy. Journal of Clinical Psychopharmacology 24:400-403.
van Waarde JA, van Oudheusden LJ, Verwey B, Giltay EJ, van der Mast RC (2012) Clinical predictors of seizure threshold in electroconvulsive therapy: a prospective study. Eur Arch Psychiatry Clin Neurosci.
van Waarde JA, Scholte HS, van Oudheusden LJB, Verwey B, Denys D, van Wingen GA (submitted) A functional MRI marker predicts the outcome of electroconvulsive therapy in severe and treatment-resistant depression.
Veroni C, Gabriele L, Canini I, Castiello L, Coccia E, Remoli ME, Columba-Cabezas S, Arico E, Aloisi F, Agresti C (2010) Activation of TNF receptor 2 in microglia promotes induction of anti-inflammatory pathways. Mol Cell Neurosci 45:234-244.
Weissman AN, Beck AT (1978) Development and validation of the dysfunctional attitude scale: a preliminary investigation. In: Paper presented at the Annual Meeting of the American Educational Research Association. Toronto.
Wium-Andersen MK, Orsted DD, Nielsen SF, Nordestgaard BG (2013) Elevated C-reactive protein levels, psychological distress, and depression in 73, 131 individuals. JAMA Psychiatry 70:176-184.
Zunszain PA, Anacker C, Cattaneo A, Carvalho LA, Pariante CM (2011) Glucocorticoids, cytokines and brain abnormalities in depression. Prog Neuropsychopharmacol Biol Psychiatry 35:722-729.

Appendix I - LIST OF BIOMARKERS

The potential biomarkers listed below will be investigated because of the indicated association with MDD or ECT-treatment (or both).

Immune system

Interleukin 2 (IL-2)

Interleukin 2 or IL-2 is a cytokine which binds to the interleukin 2 receptor, from this receptor it is known that it is elevated in major depression making IL-2 an interesting marker to study (Liu et al., 2012).

Interleukin 6 (IL-6)

Interleukin 6 or IL-6 is a cytokine with both pro as well as anti inflammatory properties. Research has shown that IL-6 has inhibitory effects on neurogenesis within the hippocampus promoting depressive behaviour. Further, it was shown that IL-6 serum levels were elevated in depressive patients compared to healthy controls indicating that IL-6 plays an role in major depression (Schmidt et al., 2011).

Interleukin 10 (IL-10)

Interleukin 10 or IL-10 is cytokine with anti inflammatory properties which plays an important role in balancing pro and anti inflammatory markers. Research showed that within major depression IL-10 levels were elevated but others showed no elevation (Liu et al., 2012). The precise role of this cytokine is still largely unknown and worth studying.

Herpes Virus entry mediator (HVEM)

Herpes Virus Entry mediator belongs to the TNF receptor super family and is also known as TNFSF14 or LIGHT (Gutierrez and Davies, 2011). Not much is known about the precise role of this receptor in major depression but preliminary results by Brainlabs BV showed that HVEM was up regulated in serum samples of depressive patients. During development HVEM is a negative regulator of BDNF promoted neuriet growth suggesting that HVEM is involved in neuroplasticity making HVEM and interesting marker (Gutierrez and Davies, 2011).

Cyclooxygenase 2 (COX2)

Cyclooxygenase 2 is an inducible inflammatory reaction promoter protein and genetic studies showed that certain mutations in the Gene encoding COX2 protect against inflammatory induced depression (Muller et al., 2011).

lipocalin 2 (LCN2)

Lipocalin 2 is a an acute phase protein mostly produced by astrocytes and microglia (Choi et al., 2011) in response to TNF receptor 1 activation (Naude et al., 2012) and is believed to be involved in brain inflammation (Choi et al., 2011). LCN2 is also an important protein in Alzheimer's, Naude et al showed that LCN2 sensitizes neurons to beta ameloid toxicity, further it blocks the protective TNF alpha 2 receptor which is believed to promote neuroprotective mechanisms. Very recently Naude et al showed that LCN2 is also an important protein in major depression, LCN2 is elevated in serum samples of major depression patients compared to healthy controls, also LCN2 levels are different between current depressive patients and patients which are in remission for 1 month (Naude et al. submitted).

C Reactive Protein (CRP)

C reactive protein is a low grade inflammatory protein and research suggested that increased CRP levels predict early signs of cognitive symptoms associated with depression (Muller et al., 2011). Further, a recent study in a large population showed that increased levels of CRP are associated with increased risks of psychological distress and depression which further underlines the importance of CRP (Wium-Andersen et al., 2013).

Myeloperoxidase (MPO)

Myeloperoxidase is a protein mainly secreted by leukocytes and microglia and is involved in the innate immune system. Upon secretion it creates reactive oxygen species increasing oxidative stress resulting in increased tissue damage during inflammation. Twin studies showed that the levels of MPO are 32% higher within depression compared to healthy individuals. It is suggested that MPO is a good marker for microglia activation (Tamatam et al., 2012). Another study showed increased mRNA MPO plasma levels within major depression suggesting also an increased MPO gene expression (Galecki et al., 2012).

Tumor necrosis factor alpha (TNF alpha)/ TNF alpha receptors 1 and 2

Tumor necrosis factor alpha is an important inflammatory protein with both protective as well as detrimental effects (Veroni et al., 2010) by acting on its receptors, TNF alpha receptor 1 and 2 (Heller and Kronke, 1994). It is most likely that TNF alpha plays an important role in

the neurodegeneration of the hippocampus, which is often observed in major depression, by inhibiting adult hippocampal neurogenesis (Schmidt et al., 2011). TNF alpha serum levels within major depressive patients is elevated compared against healthy individuals. Further, applying electro convulsive therapy significantly reduces TNF levels in the serum indicating that TNF alpha might be an interesting marker for ECT (Hestad et al., 2003).

Corticotropin releasing hormone (CRH)

Corticotropin releasing hormone is released by the hypothalamus and promotes release of ACTH from the pituatary in turn promoting release of cortisol from the adrenal glands (Zunszain et al., 2011). CRH is part of the HPA axis from which is known that it is involved in major depression.

Leptin

Leptin is an adipose secreted protein involved in food regulation (Klok et al., 2007). Studies with multiple animal models suggested that impairments in leptin production or leptin resistance are related to the development of major depression. Studies also indicate an relation between leptin levels and depression severity within humans where higher levels of leptin correlate with more severe depression but interestingly also with a higher body mass index (Morris et al., 2012).

Prolactin

Prolactin release is controlled by the hypothalamus. Prolactin levels are especially high during pregnancy and a recent study showed that prolactin increases neurogenesis in the subventricular zone of pregnant mice (Shingo et al., 2003). Further prolactin stimulates proliferation of oligodendrocytes responsible for myelination axons within the central nervous system (Gregg et al., 2007). Reduced serotonin levels within the brain of depressed patients may lead to reduced prolactin levels also reduced prolactin levels may be responsible for altered mood in females after pregnancy (Stuebe et al., 2012).

Growth factors

Endothelin growth factor 1 (EGF-1)

Endothelin growth factor 1 also known as endothelin 1 is a growth factor and plays an important in the vascular system. A study performed in 2011 showed that increased levels of endothelin 1 correlated with increased depression severity in patients with a history of coronal artery disease (Burg et al., 2011).

Brain-derived neurotropic factor (BDNF)

Brain derived neurotropic factor promotes neurogenesis within the hippocampus. Alterations in BDNF is one of the main hypothesis of major depression. Multiple lines of research have shown that stress reduces BDNF signalling and treatment with anti depressants increases BDNF (Krishnan and Nestler, 2008). BDNF serum levels are also lower in patients with major depression compared against healthy controls further underpinning the importance of BDNF (Schmidt et al., 2011). Preliminary studies in mice undergoing ECS showed that BDNF is up regulated within the hippocampus after multiple shocks.

VGF2 nerve growth factor (VGF-2)

VGF2 is an important growth factor and is involved in synaptic plasticity. Alder et al. showed that VGF expression is up regulated in response to BDNF and that VGF plays a role in synaptic strengthening (Alder et al., 2003).

Vaso-Endothelial growth factor (VEGF)

Recently it was shown that Vascular endothelial growth factor (VEGF) is increased in serum of patients treated with electroconvulsive therapy (Minelli et al., 2011). VEGF is an important growth factor and involved in angiogenesis but also in neurogenesis within the hippocampus. Studies have shown that chronic stress results in decreased levels of VEGF which can be rescued by administrating antidepressants which increases hippocampal VEGF levels. VEGF produced peripheral has also important effects on the brain which indicates that serum levels of VEGF most likely reflect brain levels (Schmidt et al., 2011).

Insulin like growth factor-1

Insulin like growth factor 1 is an important growth factor involved in many processes, within the brain it plays an important role in promoting neurogenesis. IGF-1 receptors are highly abundant in the human brain, however IGF-1 is produced peripheral mostly by the liver. Studies showed that when IGF-1 is injected within the blood stream of depressive individuals, results in a remission of the depression but also to increased neurogenesis suggesting that IGF-1 plays an important role in the aetiology of major depression (Schmidt et al., 2011).

V-erb-b2 erythroblastic leukaemia viral oncogene homolog 3 (erbB3)

V-erb-b2 erythroblastic leukaemia viral oncogene homolog 3 or erbB3 is a tyrosine kinase receptor which belongs to the epidermal growth factor receptor (EGFR) family. Studies with EGFR receptor deficient mice showed that epithelial and glial cell types are most affected by the deficiency. erbB3 is an important target of proteins belonging to the neuregulin family and

plays an important role in the formation of oligodendrocytes, initiation of cell signaling pathways for survival, growth and transformation and also implicated in the myelination process by regulating the growth and differentiation of Schwann cells. A recent study showed that erbB3 mRNA levels within leukocytes were reduced in major depressive patients which suggests that erbB3 might also be involved in pathology of major depression (Milanesi et al., 2012).

Autophagy

Tetrahydrobiopterin (BH4)

Tetrahydrobiopterin is an important cofactor in the synthesis of serotonin, dopamine and noradraline. Deficiencies in BH4 are associated with depression and other mental disorders (Koenig and Butler, 2011). Synthesis of BH4 is tightly connected to proper functioning of the immune system since interferon Y stimulates the production of BH4 (Sperner-Unterweger et al., 2012). Deficiencies in BH4 also induces autophagy which is a catabolic mechanism involving cell degradation of dysfunctional or unnecessary cell components (Lin et al., 2013). deficiencies in BH4 lead to inactivation of the mTORC1 pathway (Kwak et al., 2011).

Neuropeptides

Neuropeptide Y (NPY)

Neuropeptide Y is not considered to be a neurotransmitter but still is of great importance in the brain where it functions as a modulating neuropeptide. Genetic evolution studies showed that NPY is highly conserved suggesting that it has important functions most likely in basic physiological systems. From NPY it is known that it has multiple effects ranging from regulating food intake and modulating cognition to reducing neuronal excitability and exhibiting anticonvulsive properties. Electro convulsive shock, the animal variant of ECT applied to rats showed that levels of NPY are increased only after multiple shocks (Redrobe et al., 2002). It is interesting to study if this also applies to humans.

Viral factor

Cytomegalovirus (CMV)

Cytomegalovirus (CMV) belongs to the family of herpes viruses and can be mostly found in leukocytes. Research suggests that infection with CMV has an important effect on the ageing process increasing the change of getting an infectious disease but also increases mortality rates in the elderly (Kanapeckiene et al., 2007). Further infectious agents such as CMV are

believed to play an important role in neurodegenerative diseases such as Alzheimer's, in this light CMV and other herpes viruses were associated with cognitive impairments in elderly people (Strandberg et al., 2004). A Indian case study concerning a female patient with viral encephalitis who was totally unresponsive was treated with ECT which after 6 treatments resulted in a great improvement suggesting that ECT might also counteracts the effects of viral induced encephalitis (Shukla et al., 2012).

<u>Other factors</u>

<u>Immune cell replication</u>

All immune cells from the monocytic/B-cell lineage arise from precursor cells in the bone marrow. These precursor cells carry the CD34+ (Cluster of Differentiation) phenotype. Immediately after ECT trophic factors are released that specifically influence bone marrow replication (Stelzhammer et al., 2012). The CD34+ cell type prevalence is routinely measured in blood of patients with haematological changes due to bone marrow disorders such as myelodysplasia. The CD34+ cell type has already been shown to increase in a routine measurement from a bone marrow transplantation patient with ECT treatment for psychotic depression. CD34+ cells are capable of differentiating into microglia cells that have important immune functions in the brain (Asheuer et al., 2004).

<u>IDO activity</u>

It has been proven that the immune system is involved in altered tryptophan metabolism affecting levels of the neurotransmitter serotonin by increasing the enzyme indoleamine 2,3-dioxygenase (IDO) in for example the microglia. Increased activation of this enzyme results in the rapid depletion of tryptophan and serotonin and results in the formation of kynurenine and other toxic side products (Muller et al., 2011).

<u>Monocyte studies</u>

Monocyte studies are a part of the Moodinflame studies focussed at determine inflammation processes in affective disorders such as major depression. During our study we will study thesignature of mRNA within monocytes to investigate inflammation processes and possible effects of ECT on normalising the immune system in these cells.

Phospholipase D (PLD)

A recent study showed that PLD was down regulated in a rat depression model. From PLD it is known that it is involved in neurite outgrowth, cell growth and proliferation. Further, PLD is

essential for producing choline and phosphatidic acid (PA) from phosphatidylcholine (PC). PA is involved in controlling many basic cell functions by activating the PI3K/AKT/mTOR pathway (Feng and Huang, 2013).

Genome wide DNA methylation

Multiple studies have shown that there are differences in methylation status between healthy and depressed subjects ranging from hypermethylation to hypomethylation (Schroeder et al., 2010). Further a recent study showed that there are distinct patterns of methylation associated with different pathophysiological mechanisms of depression (Uddin et al., 2011). To our knowledge it is possible that differences in ECT treatment response and efficacy are dependent on different underlying pathological mechanisms, DNA methylation patterns may therefore be an interesting biomarker to study if DNA methylation patterns associate with changes in depression severity during and after ECT.

Appendix II - Schema-intervention

Het selecteren van depressogene cognities en situaties waarin ze optreden

Selectie van regelmatig bij patiënt voorkomende depressogene cognities vindt vooraf op de afdeling plaats door afname van de ATQ-R vragenlijst (Automatic Thoughts Questionnaire-Revisited, Kendall 1989, vertaling Raes e.a. 2001). Dit is een vragenlijst die in gereviseerde vorm naast 30 depressogene items ook 10 positieve items bevat. Invullen ervan kost ongeveer 10-15 minuten. Patiënt geeft vervolgens aan welke items de meest belangrijke rol spelen in zijn huidige depressie d.m.v. een top-6.

De depressogene items uit de top-6 worden met de onderzoeker nader besproken.
Aan patiënt wordt bij elk item gevraagd of hij een duidelijke en recente situatie voor de geest kan halen waarin deze gedachte een rol speelde die zijn stemming negatief beïnvloedde.
De situatie dient zo duidelijke en levendig mogelijk te worden geschetst. De patiënt kan geholpen worden met het beschrijven van de situatie door vragen als:

Waar was u op dat moment toen de gedachte….. door u heen ging? Wie waren er nog meer bij? Wat gebeurde er op dat moment? Of: *wat speelde er op dat moment in breder verband? Hoe reageerden de anderen? Wat zeiden ze* (zo letterlijk mogelijk)? *Hoe reageerde u? Wat deed u? Wat zei u op dat moment* (zo letterlijk mogelijk)?

Patiënt kan ook gevraagd worden zijn ogen te sluiten en zich de situatie zo helder mogelijk voor te stellen. Dit levert vaak meer details op. Details als hoe anderen die er bij waren gekleed, de weersomstandigheden op dat moment en details van de omgeving etc kunnen gebruikt worden om later de situatie en bijbehorende stemming weer op te kunnen roepen. Zoveel mogelijk zintuigmodaliteiten die patiënt zich nog kan herinneren worden er bij betrokken: zien, horen, voelen, ruiken.
Welke lichaamshouding had patiënt in de situatie? Keek hij naar de grond, teneergeslagen, met hangende schouders? Voelde hij de kracht wegtrekken uit zijn lichaam?
Welke gezichtsexpressie had patiënt op dat moment? Kan hij de lichaamshouding inclusief gezichtsexpressie voordoen?

Deze situatie met de erin optredende depressogene cognitie worden door de onderzoeker genoteerd. Zoveel mogelijk specifieke details die later voor het oproepen van de situatie direct voorafgaand aan de ECT gebruikt kunnen worden, worden hierbij aangetekend.

Wanneer patiënt geen duidelijk recent voorbeeld kan geven waarin de depressogene gedachte een rol speelde, kan hem gevraagd worden naar een duidelijk voorbeeld verder terug in de tijd waarin deze gedachte een rol speelde.

Weet u een ander moment in uw leven waarin de gedachte …. sterk door u heen ging en u zich heel somber ging voelen? Wanneer was dat? Waar was u toen? Wie waren er bij? Wat speelde er toen? Etc.

Met patiënt worden op deze wijze 6 verschillende situaties naar voren gehaald en met zo veel mogelijk specifieke details vastgelegd.

Het presenteren van situaties die verbonden zijn met depressogene gedachten

Kort voorafgaand aan de ECT wordt de eerste situatie aan patiënt gepresenteerd. De 6 verschillende situaties worden tijdens 6 verschillende ECT sessies gebruikt. Wanneer er meer dan 6 ECT sessies nodig zijn, worden de 6 situaties in willekeurige volgorde opnieuw gebruikt. Het succes van de interventie op de stemming wordt gemeten met een visual analogue scale voor en na de interventie.

Patiënt wordt gevraagd de ogen te sluiten (tenzij deze daar bezwaren tegen heeft). *U heeft mij de eerste keer verteld van de situatie….waarin u zich zo depressief voelde.*
Ik wil u vragen zo goed als lukt terug te gaan naar die situatie en dat moment waarin u zich zo somber voelde omdat u dacht ….

De details van de situatie worden vervolgens een voor een gepresenteerd. De patiënt kan geholpen worden zich de situatie weer voor te stellen door details te presenteren in de tegenwoordige tijd:

Je bent op dit moment weer terug bij… Kijk om je heen en neem de situatie goed in je op. Bekijk ook de anderen die er zijn.

Ook de depressogene gedachte wordt ten minste drie maal aan patiënt gepresenteerd:

En terwijl je daar bent en dat alles ziet denk je…. En met die gedachte voel je je je somberheid. Je bent helemaal overtuigd van het idee dat … Probeer dat gevoel zo duidelijk mogelijk te voelen, samen met de gedachte….

Ook lichaamshouding en de gezichtsexpressie die samengaan met de verlaagde stemming in de situatie kunnen worden aangemoedigd:

Voel hoe teneergeslagen je je voelt. Je schouders gaan hangen. Je voelt je futlozer worden. Je wordt kleiner. Laat die gevoelens toe in je lichaam.

Presentatie van de cognities en de stemming worden afgewisseld met details uit de situatie. Het tempo hierbij is rustig. Er wordt regelmatig enkele tellen gepauseerd om patiënt de gelegenheid te geven zich de situatie voor te stellen en de stemming en gedachten naar voren te laten komen.

Af en toe wordt patiënt gevraagd of hij op dit moment ook de negatieve stemming voelt opkomen. *Voelt u de somberheid?*

Wanneer patiënt dit beaamt wordt hij hierin aangemoedigd: *prima, blijf bij dat gevoel en bij die gedachte….*

Wanneer patiënt nee schud wordt hij aangemoedigd zich de situatie zo goed mogelijk te blijven voorstellen en de gedachten te blijven herhalen: *ok, blijf u de situatie zo goed mogelijk voorstellen en de gedachte…..*
Achteraf kan besproken worden wat het oproepen van de situatie heeft gehinderd.

Na 5-10 minuten wordt het oproepen van situatie en de daar gekoppelde gedachten en stemming afgerond.

Om de oefening af te ronden wil ik u vragen om terug te keren naar het huidige moment. Als u zo ver bent kunt u uw ogen openen.
