# Supplementary material for: Effectiveness of Emotional Memory Reactivation vs Control Memory Reactivation Before Electroconvulsive Therapy in Adult Patients With Depressive Disorder: A Randomized Clinical Trial
Source: JAMA Netw Open. 2020 Aug 4;3(8):e2012389. doi: 10.1001/jamanetworkopen.2020.12389 (PMC7403919; doi:10.1001/jamanetworkopen.2020.12389)
Supplement: Supplement 2. — Data Sharing Statement [file jamanetwopen-3-e2012389-s002.pdf]

Data Sharing Statement

Scheepens. Effectiveness of Emotional Memory Reactivation vs Control Memory Reactivation Before Electroconvulsive Therapy in Adult Patients With Depressive Disorder. *JAMA Netw Open*. Published August 04, 2020. 10.1001/jamanetworkopen.2020.12389

Data
Data available: Yes
Data types: Participant data with identifiers
How to access data: g.a.vanwingen@amsterdamumc.nl
When available: With publication

Supporting Documents
Document types: None

Additional Information
Who can access the data: researchers whose proposed use of the data has been approved
Types of analyses: for a specificeer purpose
Mechanisms of data availability: after approval of a proposal
Any additional restrictions: none
